# What if eye...? Computationally recreating vision evolution

**DOI:** 10.1126/sciadv.ady2888

**Published:** 2025-12-17

**Authors:** Kushagra Tiwary, Aaron Young, Zaid Tasneem, Tzofi Klinghoffer, Akshat Dave, Tomaso Poggio, Dan-Eric Nilsson, Brian Cheung, Ramesh Raskar

**Affiliations:** ^1^ Camera Culture, MIT Media Laboratory, Cambridge, USA.; ^2^ Computational Imaging Laboratory, Rice University, Houston, USA.; ^3^ Charles Stark Draper Laboratory, Cambridge, USA.; ^4^ Center for Brains, Minds, and Machines, MIT, Cambridge, USA.; ^5^ Lund Vision Group, Lund University, Lund, Sweden.; ^6^ InfoLab, MIT CSAIL, Cambridge, USA.

## Abstract

Natural selection has produced diverse vision systems, from simple patches of photoreceptors to complex camera eyes, representing just one set of evolutionary outcomes. Computational evolution offers a way to systematically test hypotheses, isolate individual factors, and ask the “why” questions behind vision. We recreate vision evolution by coevolving eyes and behaviors in embodied agents and use this to illuminate principles shaping vision across different levels of the Marr’s hierarchy. This leads to three key findings: First, we provide computational evidence that task-specific selection drives bifurcation in eye evolution. Second, we reveal how optical innovations naturally emerge to resolve fundamental trade-offs between light collection and spatial precision. Third, we uncover scaling laws between visual acuity and neural processing that provide insights into long-standing hypothesis behind eye and brain size. Our work introduces a paradigm that uses embodied artificial intelligence (AI) as hypothesis-testing machines that can help accelerate discoveries in vision science.

## Teaser

Recreating evolution by coevolving eyes and behaviors in embodied agents allows exploration of what-if questions in vision.

## INTRODUCTION

What if vision were only used for navigation or detection? What if eyes never evolved optical elements like lenses? What if animal brains stayed small throughout evolution? Operating over millions of years and culminating in millions of unique perception systems (*1*), natural evolution has followed specific evolutionary trajectories in its development of vision. What if there was a tool to simulate alternative paths that evolution did not take? By computationally recreating the evolutionary dynamics (i.e., mutation, selection, adaptation), which gave rise to the remarkable diversity of eyes we see today, we can explore different evolutionary trajectories and systematically probe the principles that shape visual diversity. This framework would enable us to test hypotheses about the relationships between eye morphology, neural processing, and environmental pressures, and guide the design of previously unexplored vision systems for artificial agents both in nature and engineering.

**Fig. 1.**
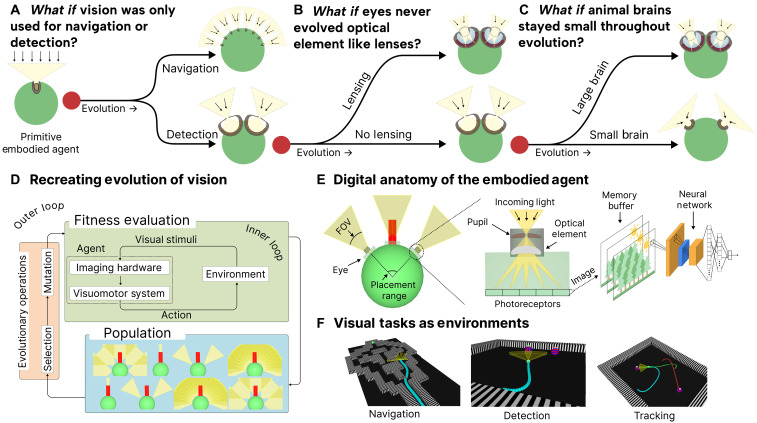
Computational evolution of embodied AI agents reveals how environmental pressures shaped natural vision evolution. We evolve artificial embodied agents to show how three evolutionary branch points shaped vision evolution. We use our framework to understand (**A**) how environmental specificity led to distinct eye morphologies, (**B**) how optical elements emerge when embodied agents evolve to discriminate between objects while accounting for physical trade-offs of light collection versus spatial precision in an environment, and (**C**) how visual task error scales as power law with visual acuity and number of parameters revealing that poor visual acuity creates a fundamental bottleneck that cannot be overcome by simply scaling neural capacity. (**D**) Our framework mirrors natural selection: An outer loop governs genetic inheritance and selection over evolutionary timescales, while an inner loop enables agents to learn through sensory feedback (lifetime adaptation). This nested structure reflects the Baldwin effect (*46*), where agents better able to learn throughout their lifetime are more likely to be selected, accelerating evolutionary adaptation of their specific morphologies. (**E**) The agent’s digital anatomy parallels biological visual systems: from eye morphology and placement, through optical elements and photoreceptors (mimicking retinal organization), to neural processing (analogous to visual cortex). (**F**) Agents evolve to solve three distinct visual tasks to probe how environmental pressures shape vision: (i) Navigation: orientation and obstacle avoidance through a maze-like environment; (ii) Detection: object discrimination between a goal object (food) and adversarial object(s) (poison); and (iii) Tracking: identical to Detection, but the objects move. Our results highlight how embodied agents can serve as scientific instruments to understand biological visual intelligence.

In this work, we introduce a framework to elucidate how environmental pressures shaped visual system evolution using embodied artificial intelligence (AI). Our approach evolves the eyes and neural circuitry of embodied agents inside physics-based simulation environments to understand what environmental factors drove vision evolution. While comparative biology has revealed remarkable insight into vision evolution (*1*–*3*), testing causal hypotheses or answering “what-if” questions has been difficult as these will typically require rerunning evolution to understand whether the effect still occurs by first performing an intervention on the possible cause (*4*) (Fig. 1). Our work builds on two foundational directions: first, pioneered by Grey Walter’s machina speculatrix (*5*, *6*), the use of evolutionary robotics to test scientific hypotheses about biological mechanisms and processes (*7*–*11*). Through learnable embodied agents and a computational model for biological phenomena, we can study predator-prey dynamics (*12*), brain-body coevolution (*13*), environmental adaptation (*14*–*18*), or “why” questions in neuroscience (*19*) such as the emergence of foveal image sampling (*20*) or peripheral vision (*21*). However, vision evolution has yet to be studied in this context. Second, our work builds on the emergence of deep reinforcement learning (DRL) as a powerful tool for discovery in domains that can be formulated as reward-driven games (*22*–*25*). Our work illuminates evolutionary principles shaping vision by creating single-player games with specific environmental conditions that embodied agents solve by evolving their vision and learning complex behavior simultaneously. We demonstrate that visually capable embodied agents trained via DRL can serve as next-generation hypothesis-testing machines.

**Fig. 2.**
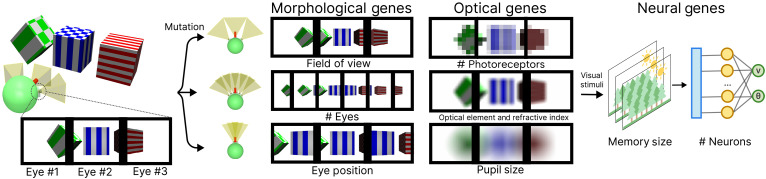
Our genetic encoding enables vision to evolve computationally. Our encoding mirrors the natural separation between sensory and neural development through three gene clusters. Morphological genes determine agent properties relating to spatially sampling the environment such as eye placement and FOV. Optical genes determine agent properties relating to how each eye interacts with incoming light in a physically plausible way, including the number of photoreceptors, optical elements, and pupil size. Neural genes describe the behavior learning capacity of the agent. These independently mutable genes enable the computational exploration of evolutionary pathways that mirror those in natural vision evolution.

We first implement a genetic encoding that unifies physical eye morphology, eye optics, and neural processing (Fig. 2), and then computationally mimic the evolutionary process in embodied agents by evolving this genetic encoding to best complete a visual task (Fig. 1D). Our framework computationally simulates hypothetical evolutionary trajectories of visual systems, enabling the coevolution of eyes and behaviors in response to environmental pressures. We use this framework to study the emergence of visual capabilities documented across animal phylogeny (*2*, *26*). Our encoding integrates morphological, optical, and neural components into a unified genome capable of describing over 10^20^ unique configurations and provides a continuous space for exploring evolutionary pathways (i.e., lens-less cup eyes, camera-type eyes, eyelet eyes, compound eyes). Subsequently, over generations, agents’ genes are selected and mutated, leading to the emergence of complex eyes and behaviors for specific visual tasks. This computational survival-of-the-fittest provides a simplified model to explore the interplay of variation and selection that shapes biological vision.

Through targeted computational experiments, our framework suggests plausible causal links between specific visual functions (*26*–*28*) and solutions, validates aspects of eye evolution as trade-offs between light collection and spatial precision (*26*, *29*), and studies relationships between eyes design, neural processing, and visual tasks. Concretely, our scientific contributions demonstrate how computational evolution can generate and test hypotheses about the emergence of visual capabilities: (i) Our framework shows that by strictly changing the visual task an agent is subject to, orientation (Navigation) task versus object discrimination (Detection) task (*26*, *28*), computational evolution produces a bifurcation in our evolved agents between camera-type and distributed eyelet-type eyes (Fig. 1A), suggesting that task-specific pressures may drive visual system divergence; (ii) our simulations demonstrate that the emergence of optical structures, such as focused lensing from primitive simple eyes (*29*), is a key innovation that can address the fundamental trade-off between light collection and spatial precision (Fig. 1B), providing computational support of this evolutionary hypothesis; (iii) we reveal sensory acuity and neural capacity scale as a power law, where decreasing task error requires complementary improvement in both dimensions—a pattern consistent with observations made in animal vision and, more recently, deep learning models used in AI (*30*, *31*), suggesting that this relationship may be a general principle of scaling across a broad scope of various types of morphologies and architectures. In addition, our engineering contributions are (i) a framework that evolves vision and behavior of embodied agents through genetic algorithms and DRL in a custom simulation framework and (ii) a genetic encoding scheme for vision that describes a diverse set of eyes and cognitive capabilities in addition to being physically based and realizable.

Due to biological complexity and computational tractability, we scope our work to recreate the system-level process of vision evolution rather than an imitation of its historical timeline. Because our goal is to understand the mechanistic principles driving vision evolution, we computationally recreate essential elements that shape natural vision. We model key components universal to biological evolution (Fig. 1D) and agent’s anatomy (Fig. 1E), and consult biological studies (*28*, *32*) when designing the simulated environments (Fig. 1F). In addition, when studying optical transitions, we use widely adopted physics-based approximations (fig. S11), a dynamics engine (*33*) for interaction, and train our agents only through sensory feedback using reinforcement learning. While our computational framework represents an approach to exploring vision evolution, fundamental limitations remain. For example, complex biological phenomena are poorly understood, such as eye genomics and development (*34*, *35*), biophysical models (*36*), or mechanistic neural circuits (*11*). Moreover, modeling evolutionary dynamics is often akin to modeling a chaotic system (*37*) as eye adaptations are often a result of many interdependent pressures. However, our results highlight how embodied agents trained via DRL can serve as scientific instruments to understand biological visual intelligence.

## RESULTS

Our computational framework tests hypotheses about how specific environmental pressures shape eye morphologies, neural architectures, and behavior. While previous work has used evolutionary algorithms to independently design optical systems (*38*, *39*), embodied agent morphologies (*7*, *40*, *41*), or visually guided behaviors (*42*), our approach uniquely evolves embodied agents with both eyes and behaviors together. This enables the automatic task-driven discovery of diverse vision-based embodied agents.

### A what if ...? world

We model the world in which embodied agents interact as a DRL environment, where agents must evolve appropriate vision capabilities such that they can learn effective behavior. It is infeasible, however, to model all factors that contribute to the evolution of vision; thus, we model each environment as corresponding to a specific function of vision, such as orientation or object discrimination. In this way, each environment represents a single task that models the functional pressures hypothesized to drive the emergence of vision. In the Discussion section, we discuss extension to a multitask and multiagent setting that reflects real-world environments.

We model three distinct tasks to isolate their effects on the evolution of vision (*28*, *43*): (i) Navigation: orientation and obstacle avoidance in a maze-like environment; (ii) Detection: discriminating between a goal object (food) and one or more adversarial objects (poison); and (iii) Tracking: discriminating with the same objects, but both objects move at a fixed speed with random headings, so the agent must compensate for motion—track the food and evade an approaching adversary. Each task serves as a simplification of real-world environments to study fundamental aspects of vision; the environmental appearance reflects common approaches in vision science to isolate specific vision-based behaviors (*32*).

We create these tasks in a MuJoCo simulation environment (*33*, *44*) with added support for physics-based imaging through programmable lenses and aperture-controlled retinal models, enabling complex imaging and evolutionary search. Each agent in this environment is modeled as a point mass (the green sphere in Fig. 2) with a heading and forward velocity. We refer the reader to Materials and Methods for technical details such as complete reward functions that incentivize these visual behaviors.

### Genetic encoding for vision

Vision in nature has coevolved as a function of sensing and the underlying neural circuitry, subsequently constraining behavior that an animal learns during its lifetime (*26*, *43*, *45*). At a population scale, this continuous feedback loop between evolution and learning is similar in nature to the Baldwin effect (*46*), where the ability to learn specific behaviors increases the likelihood of a selection of genetic traits for future generations. Similarly, we create a genotype that directly encodes both the physical (morphological and optical) and the neurological components of an agent’s vision. While we use genetic terminology for consistency with evolutionary and computational literature, our encoding directly represents phenotypic traits (morphology, optics, and neural architecture) rather than gene-like units that would polygenically determine these traits in biological systems. This simplified phenotype-to-performance mapping allows us to directly study how visual morphology influences behavioral outcomes. Rather than incorporating neural network weights directly in the genomic encoding (*47*–*49*), we train each agent from scratch to learn behaviors specific to the mutated eye design.

Similar to nature, our genetic encoding scheme needs to be general enough to allow the emergence of a diverse set of eyes and cognitive capabilities while being physically realizable. Therefore, we first conceptualize an agent’s eye as a physical sensor that converts photons into neural impulses. These impulses are then followed by cognition, which enables agents to interact within the environment. We categorize the genotype into three subgenes (Fig. 2): morphological genes that control morphological features such as placement, quantity, and field of view (FOV); optical genes that control features about the captured image; and neural genes that control the agent’s behavior. Each gene describes a subset of an agent’s visual system and is mutated through specific evolutionary operators (Fig. 2). The exact genetic encoding is discussed in more detail in Materials and Methods.

### Coevolution of vision and behavior

Our approach computationally simulates nature’s coevolution approach to vision innovation: Changes in sensory capabilities directly influence behavioral performance, which, in turn, guides the evolution of future eye morphologies, optics, and behaviors. We implement coevolution through two nested loops that mirror the interplay between evolutionary timescales and lifelong adaptation. Over generations, the outer evolutionary loop uses the covariance matrix adaptation evolutionary strategy (CMA-ES) (*50*, *51*) to enable efficient selection and mutation of populations of agents. Within each generation, the inner learning loop, an agent with the selected genotype, is instantiated and trained to solve a visual task through reinforcement learning via proximal policy optimization (PPO) (*52*). During the agent’s lifetime, performance is evaluated within the same visual task. The fitness of each agent is then used in the following generation to selectively adapt the population’s genotypes. By varying the random seed of the inner and outer loops of this framework, we demonstrate consistency across evolutionary runs and limit noise effects for specific reinforcement learning (RL) level training loops. We discuss the evolution and learning loops in more detail in Materials and Methods.

### What if the goals for vision were different?

Understanding how specific visual tasks shaped the evolution of eyes remains a major challenge because animals are required to solve multiple visual tasks simultaneously. For instance, honeybees have evolved compound eyes with around 5000 individual receptors, balancing trade-offs between extracting optic flow to maintain equidistance from obstacles and regulate flight speed, and sufficient spatial resolution to discern body movements of other bees in their colony (*32*). This coupling of tasks in nature makes it difficult to understand how individual visual demands influence eye evolution. For instance, dragonflies have evolved compound eyes with high-resolution regions, making it challenging to identify which visual adaptation was a result of which environmental pressure. Thus, would evolution converge on similar eye morphologies as found in nature if we could isolate individual visual tasks? To address this, we create two distinct environments that isolate specific visual demands, allowing us to observe how eye morphologies evolve when optimizing for a single task.

Our computational framework tests vision evolution through two distinct tasks that isolate different environmental pressures. The Navigation task is a goal-less orientation task (*28*) where agents are incentivized to traverse a maze environment as fast as possible while avoiding collisions with walls and forward barriers that alternate with black-and-white striped patterns of different frequencies [similar to navigational setups that test navigational abilities within honeybees (*32*)]. Conversely, the Detection task is an object discrimination task where agents choose the goal sphere between three visually similar spherical objects in an open environment (this can be conceptualized as identifying food from poison with the only difference being the rotation of a high frequency spherical pattern on the sphere). In both tasks, agents control only their forward speed and heading. Agents in both tasks are initialized by generating a population via randomly mutating the genotype of a primitive agent (a single eye with one photoreceptor and an FOV of 45°). Over the course of evolution, agents mutate by using only morphological constraints: adding or removing photoreceptors, adding or removing eyes, and adjusting the eye’s placement. As the Navigation task is essentially two-dimensional (2D), only photoreceptors along the horizontal width of the eye are added or removed. We compare the resulting imaging systems of the optimized agents using cycles per degree (CPD) (*31*, *53*, *54*), which is a measure of the spatial frequencies observable to the agent; a higher CPD value corresponds to a better ability to resolve fine spatial details in a scene (*31*) (we describe CPD in more detail in the Supplementary Materials.)

**Fig. 3.**
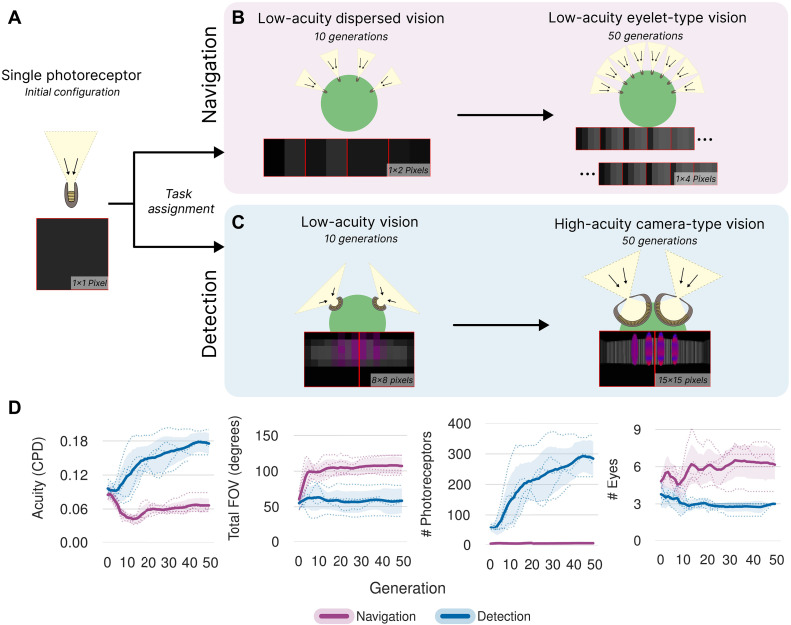
Low- and high-acuity spatial tasks lead to distributed and camera eyes, respectively. (**A**) We initialize a population of agents for two visual tasks (Detection and Navigation) with a single eye with one photoreceptor. We then evolve a population of agents subject to morphological mutations: add photoreceptor, add eye, and adjust placement. (**B**) In the Navigation task, we first observe an emergence of dispersed vision, where many eyes are used. Some agents in early generations develop compound-eye configurations with multiple single-photoreceptor eyes distributed around the body. By generation 50, most agents evolve into an eyelet-type vision system consisting of seven individual eyes, each with four photoreceptors (4 by 1 resolution), distributed over the entire diameter of the agent. This resembles the visual architecture found in strepsipteran insects (*55*) rather than typical compound eyes. (**C**) In the Detection task, we initially observe the emergence of low-resolution vision. After 50 generations, the population has converged on a morphology consisting of two forward-facing, high-resolution camera-type eyes each with 225 photoreceptors (15 by 15 resolution). (**D**) Configuration versus generation plots are shown, depicting the evolutionary progression of the mean agent in the population and the task dependence on evolutionary adaptation. For each task, we run five trials varying the random seed for CMA-ES. The plots show the mean across all trials as a solid line, the mean of each run as dotted lines, and the 95% bootstrapped confidence interval across all trials as shaded regions.

From an initial configuration of one eye with a single photoreceptor, agents evolve distinctly different morphologies under each task. To enforce realistic physical constraints, we limit the allowed configurations to prevent overlap based on photoreceptor size and eye radius. For the obstacle avoidance demands of high-speed navigation, our Navigation-specialized agents initially evolved multiple single-photoreceptor eyes distributed around their heads—similar to a compound eye configuration found in typical insects (*32*). However, around generation 15, many of these agents subsequently evolved to add more photoreceptors per eye while reducing the total number of eyes, ultimately converging on seven widely distributed visual units, each containing four photoreceptors (1 by 4) (Fig. 3B). The final configuration optimizes full-body coverage with a 135° total FOV, enabling rapid environmental sampling during high-speed maze traversal. In contrast, Detection-specialized agents develop two forward-facing eyes with 225 photoreceptors (15 by 15) each (Fig. 3C), concentrating their visual resources frontally. This evolutionary divergence reflects task-specific optimization: Navigation agents maximize spatial awareness through distributed low-resolution sensing, while detection agents sacrifice peripheral vision for enhanced frontal acuity, enabling object discrimination at greater distances within their fixed time constraint.

The divergence from single pixel ommatidia to multiphotoreceptor units is notable, resembling the unusual “eyelets” of strepsipteran insects (*55*). In traditional compound eyes, each ommatidium contributes a single photoreceptor, or pixel, to the visual mosaic, whereas eyelets group multiple photoreceptors within one unit. This increases image-forming capacity within each ommatidium. In our obstacle-avoidance task, eyelet eyes achieved higher fitness than single-pixel compound eyes, suggesting advantages of chunk-based vision as an alternative evolutionary strategy (ES). Our agents’ evolution of eyelets likely reflects the absence of modeled effects (e.g., neural pooling, spectral comparisons, and geometric packing) that likely lead to the success of compound eyes.

Our agent’s morphological divergence directly influences the topology of their neural network used to learn task behavior. A distributed eyelet eye configuration in our genetic scheme enables parallel processing of visual information—each additional eye gets its own small visual processing unit implemented as a Multi-Layer Perceptron (MLP) that handles processing of a specific part of the visual field before the low dimensional features from each eye are concatenated. Conversely, in the Detection task, agents evolve camera-type vision (two to three forward-facing eyes) with larger input arrays (15 by 15 by 3) per eye.

Our computational isolation of visual tasks reveals fundamental patterns that parallel natural evolution (*1*, *50*, *56*–*58*). The emergence of distinct morphologies from identical starting conditions demonstrates how environmental demands can drive visual specialization. Our navigation agents evolved distributed eyelet-based vision for efficient environmental sampling, eventually converging to a solution like strepsipteran vision (*55*) rather than typical compound eyes (*32*). When quantifying visual capabilities using CPD measurements (*31*, *53*, *54*), we found a clear trade-off between spatial coverage and resolution that mirrors natural systems (Fig. 3D). This trade-off manifests across species (*3*), where animals evolve either enhanced acuity or broader FOVs based on their ecological needs. Our simulation results provide counterfactual evidence that task-specific selection pressures can drive the emergence of these distinct visual and neural processing strategies.

### What if eyes could bend light?

Early visual systems faced a fundamental trade-off between light collection and acuity, progressing from simple light-sensitive patches to cup-shaped eyes with smaller apertures (*59*, *60*). While decreasing the size of the aperture and creating pinhole-like designs is a straightforward way to improve image formation, they severely limit light collection. This trade-off creates a performance ceiling, where further improvements in spatial resolution through pinhole designs are limited by the lack of light. This inherent limitation ultimately restricts the visual capabilities of such systems, causing a saturation in performance. We see this manifested in our results where agents with pinhole eyes plateau in fitness and cannot achieve the performance benefits of improved spatial resolution. What if we introduced optical elements that can redirect light into our agents? Lenses emerged as an innovation in biological evolution, and we investigate the impact of enabling optical elements capable of bending light in our framework.

To isolate optical evolution, we restrict mutations to the optical subspace: pupil size, optical element, and refractive index. We fixed the remaining morphological genes to the parameters evolved in the Detection task in Fig. 3. Pupil size controls the signal-to-noise ratio (SNR) by controlling the total light collection on the retina; since the noise in the environment is fixed, SNR decreases as pupil size decreases. The optical element is represented as a 2D array that can be programmed into lenses of different shapes [modeled as a diffractive optical element (DOE)] (*61*–*63*). Refractive index controls the bending of light within the optical element. These three parameters are general enough to represent a large optical design space. We discuss these parameters, the physics-based rendering model, and their relation to real eyes in Materials and Methods.

**Fig. 4.**
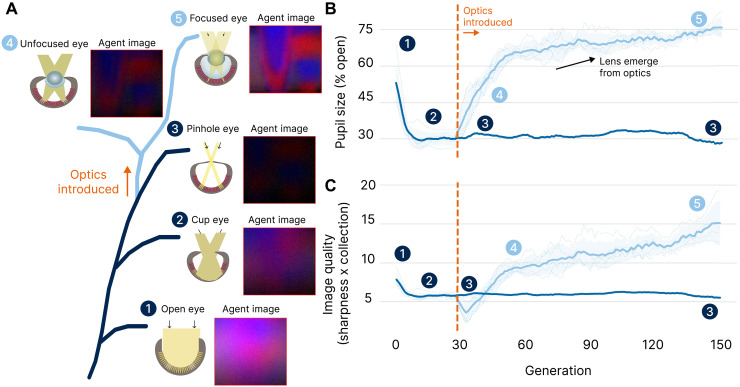
Computational evolution reveals how lensing resolves a fundamental trade-off in vision. We demonstrate that to achieve maximum fitness in the Detection task, evolution learns to evolve optical structures that balance spatial precision (optical resolution of the eye quantified using MTF) against maximizing light collection. (**A**) The evolutionary sequence shows five key stages of eye development: (1) open pupil with maximum light collection but poor spatial precision, (2) cup eye and (3) pinhole eye that achieve better spatial precision by reducing pupil size at the cost of light collection, followed by the emergence of (4) unfocused lens-based eyes, where a diffuse lens with low or uniform refractive index does not yet concentrate light effectively, and (5) focused lens-based eyes that maintain spatial precision by evolving optical structures while allowing larger pupils for more light collection. Agent images show the scene as perceived at each stage of evolution using the evolved optical genes (pupil, lens, and aperture). The ground truth scene is shown in fig. S8. (**B**) Without optics (dark blue), pupil size decreases to improve precision, sacrificing the SNR. When lensing is enabled (orange line, generation 30), larger pupils emerge as lenses evolve to maintain precision while increasing light collection. (**C**) The Image Quality metric (image sharpness × light collection) quantifies this trade-off resolution: Pinhole eyes (3) plateau at low values due to limited light collection, whereas lens-based eyes [(4) and (5)] achieve higher quality by combining good spatial precision with larger pupils. This mirrors the evolutionary pressure that drove the emergence of biological lenses, which enabled enhanced vision across lighting conditions. The dark line and shaded region show the mean across five independent evolution runs and the 95% bootstrapped confidence interval, respectively. The dotted lines represent the mean across each run.

We set out to explore how introducing optical elements might change the evolutionary trajectory of our agents’ vision in the Detection task. To do this, we designed two distinct experiments. In the first, Eye without optics, we allowed only the pupil size to mutate while keeping other factors fixed. This setup mirrored the early stages of eye evolution, where organisms could merely evolve their aperture over generations to balance light collection and spatial resolution. Across generations, a clear progression emerged: Agents began with fully open apertures [Fig. 4A (1); 1 corresponds to the open eye configuration in Figure 4A] that captured plenty of light but produced blurry images; they next converged on cup-shaped eyes [Fig. 4A (2)], trading some brightness for reduced blur; and, ultimately, they settled on near-pinhole eyes [Fig. 4A (3)] that offered sharper vision at the cost of even lower light collection. Pinhole eyes are rare in nature, found for example in the chambered nautilus and a few terrestrial spiders. These eyes typically occur in dim reef or low-light terrestrial habitats where motion is slow, and photon noise is tolerable (*43*).

After 30 generations in this baseline setting, we initiated a “counterfactual” experiment, Eye with optics, where agents could also mutate their optical element and refractive index. The sudden introduction of random lens shapes initially caused a dip in performance. However, between generations 30 and 50, evolution steadily refined these diffuse structures into slightly convex shapes [Fig. 4 (4)], showing the first signs of lens-like focusing. Beyond generation 50, agents reached a turning point: The optical element evolved into well-defined lenses with symmetric point spread functions (PSFs), while still retaining relatively large pupils [Fig. 4 (5)]. As a result, these agents escaped the tight trade-off imposed by the pinhole design, maintaining a crisp image while collecting more light, leading to higher fitness and more consistent goal discrimination. We quantify these improvements with an “Image Quality” metric, defined as the product of spatial precision (quantified by the modulation transfer function (MTF) of the PSF, which captures the system’s ability to preserve spatial frequencies) and maximum light collection. For further details on the fitness trajectories, peak signal-to-noise ratio (PSNR)/structural similarity index measure (SSIM) analyses, and 3D visualizations of the final lens designs, refer to Supplementary Text.

In addition, Eye without optics and Eye with optics fitness trajectories (fig. S2) reveal a stark contrast in detection capabilities: Whereas Eye without optics agents achieve only sporadic goal detection through random encounters, Eye with optics agents evolve reliable detection strategies by generation 130, with top performers consistently identifying multiple goals among the adversarial objects. Figure S2 also plots immediate and steady improvements in both PSNR and SSIM. Figure S4 shows how high-performing agents (*F* > 24.4) evolved singular peaks and smoother optical responses in their optical elements, whereas poor performers (*F* < 3.4) developed fragmented and multipeaked patterns. This is also reflected in the dispersed and compact PSFs of low- and high-performing agents respectively (fig. S1).

Our results demonstrate a critical sequence that provides computational insights into why lenses may have emerged over the course of vision evolution. While our agents were simply tasked with discriminating between similar-looking objects, it required the populations to evolve effective eyes subject to the fundamental trade-off in vision evolution: balancing spatial precision (needed to discriminate similar objects) with light collection (needed for reliable vision). Our image quality metric, which combines MTF-derived spatial precision with light collection, quantifies this trade-off directly. In Eye without optics, where only pupil size could vary, spatial precision saturates as pinhole eyes sacrifice light collection for acuity, resulting in dark, noisy vision. Eye with optics reveals how lens evolution resolves this constraint: DOEs evolve into lenses that maintain spatial precision while allowing larger pupils, eliminating the precision versus light collection trade-off. Our counterfactual experiment suggests that without this innovation, accurate vision would have been restricted to high-light conditions; for example, while pinhole eyes can technically produce sharp images, they are rarely found in nature due to the loss in light collection. This is in contrast with camera-type eyes in vertebrates that decouple acuity from light collection by using refractive optics and larger pupils reflecting the tension that our metric captures. This also aligns with proposed explanations that suggest that lens development coincided with an increase in eye size (*59*, *60*). The lens thus represents a fundamental innovation in the evolutionary solution space, discovered by our agents not through direct optimization of optical properties, but through the demands of achieving accurate perception on a specific behavioral task.

Our choice of optical gene parameters is based on known optical principles and maintains biological plausibility and computational tractability. While the exact physical processes that led to biological lens emergence remain unclear, one hypothesis is that lenses emerged through gradual increase in optical densities of vitreous humor present inside chambered eyes (*29*). This increased the refractive index and eventually converged to a smooth focused lensing. We abstract away this complexity by directly optimizing optical genes instead of parameters to a fluid dynamics simulation.

### What if the brain became larger?

Natural studies extensively document correlations between brain size and eye size across various species, yet they are limited to observational analysis and can only hypothesize underlying causes without definitive testing (*53*, *64*–*67*). Evolution inherently optimizes traits, discarding suboptimal configurations before they can be studied, leaving counterfactual scenarios unexplored.

**Fig. 5.**
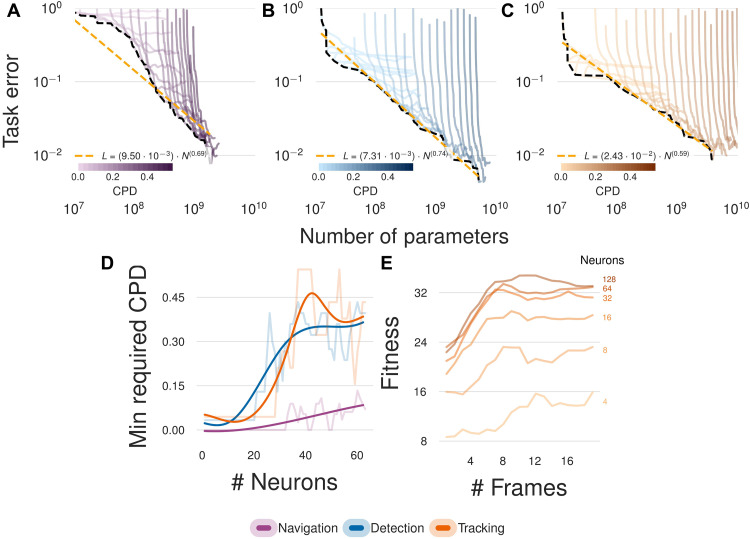
Task-dependent scaling laws reveal how visual acuity bounds performance and how temporal memory compensates for neural capacity. (**A** to **C**) Our experiments reveal visual task-dependent power-law scaling between number of parameters and visual acuity (CPD). This demonstrates that scaling in sensory input is required for embodied tasks to avoid a bottleneck that cannot be overcome by neural scaling alone. (**D**) A minimum required visual acuity compared to number of parameters for different embodied tasks suggests a hierarchy in the emergent behaviors depending on the task. (**E**) Temporal processing shows complementary scaling with neural capacity, where increased temporal memory (# Frames) can compensate for reduced neural processing, which is particularly evident in tasks with larger networks. Together, these results quantify how visual intelligence emerges from the interplay between sensory, neural, and temporal capabilities.

Our computational framework uniquely enables the exploration of these counterfactual scenarios, testing hypotheses about how scaling brain size (neural network size) alongside sensory capabilities affects visual intelligence. Our neural network parameters represent a computational analog to biological neural capacity, directly reflecting the studies showing strong correlations between brain volume and total neuron count across species, humans, and mice brains (*68*–*70*). While biological brains scale through additional complex mechanisms such as changes in neuron density, myelination patterns, and circuit specialization, we focus on the core relationship between brain size and neuron count and study scaling laws for visual intelligence. Thus, we systematically vary visual acuity of the agent eyes (CPD), the agent’s neural network size processing the visual input, and temporal memory to quantify their interplay and effect on task-specific performance in embodied agents (Fig. 5).

We uncover distinct power-law scaling relationships between neural capacity and task performance across navigation, detection, and tracking tasks. Increasing neural parameters predictably improves performance [$L=\left(9.50\cdot 10^{-3}\right)\cdot N^{0.69}$ for navigation, $L=\left(7.31\cdot 10^{-3}\right)\cdot N^{0.74}$ for detection, and $L=\left(2.43\cdot 10^{-2}\right)\cdot N^{0.59}$ for tracking], but only when visual acuity is scaled correspondingly. Limited visual acuity imposes a performance ceiling that neural scaling alone cannot surpass, highlighting a fundamental bottleneck analogous to scaling laws observed in AI systems constrained by model size, computational resources, and data availability.

Our findings identify clear transition points (Fig. 5D), where further enhancements in visual acuity provide diminishing returns for a fixed neural network size. Tasks vary in their acuity thresholds, revealing a hierarchy of visual processing demands, with the detection task requiring notably higher minimum visual acuity compared to navigation or tracking.

In addition, our experiments also demonstrate that increased temporal memory can partially compensate for reduced neural processing capacity (Fig. 5E). In tasks reliant on temporal information like tracking, extended temporal integration can achieve comparable task performance to sophisticated single-frame analysis enabled by larger neural networks. In nature, early-stage retinal processing has also been observed, such as temporal filtering and motion-opponent channels (*71*–*73*), which compress information before being processed by the brain. Such preprocessing mechanisms may improve the efficiency of our observed scaling relationships by reducing information bottlenecks.

Together, these results present a quantitative hypothesis for how visual intelligence emerges through the coordinated scaling of sensory, neural, and temporal capabilities. Concretely, we demonstrate that more effective downstream task performance is enabled by imaging the environment with higher acuity and larger processing budget. Our counterfactual approach uniquely quantifies the interdependence of processing capability and visual acuity, addressing longstanding observational hypotheses with computational evidence (*53*, *67*).

Concurrent work with comparative studies of real animals synthesizing anatomical, behavioral, and evolutionary data also show that species with high-resolution vision devote substantially more neural resources to visual processing (*43*). This further suggests coordinated scaling of eye and network size in support of visually guided behaviors. While AI systems focus on scaling neural, data, and computation capacity (*30*, *31*, *74*–*77*), our findings suggest that evolution of embodied intelligent behavior (biological or artificial) may require coordinated scaling of visual acuity and processing.

These scaling laws indicate a functional divide that manifests across animal phylogeny: Species with sophisticated object discrimination (vertebrates, arthropods, and cephalopods) have large brains, whereas those with nonobject vision (ragworms, snails, flatworms, and jellyfish) retain simple neural architectures that exploit light-field cues. Our results quantitatively capture this principle, showing that DETECTION tasks require notably higher visual acuity and neural capacity than NAVIGATION tasks. Natural evolutionary arms races further intensify these demands as prey and food sources become increasingly difficult to detect and discriminate.

## DISCUSSION

Similar to natural evolution, we follow a function over form approach, where we code the desired function through fitness and let evolutionary search discover a variety of forms that are optimal for the fitness. This results in our agent’s form (eye design and learned behavior) to emerge solely from functional pressures from the environment such as orientation, obstacle avoidance, or object discrimination. The emergent features resemble principles of real biological evolution. These results affirm our central claim that embodied agents trained with reinforcement learning can serve as hypothesis-testing machines for vision and vision evolution. The evolutionary outcomes we present are a result of the coevolution of vision-hardware (physical eye morphology and structure) and software (learned behavior of the agent). Last, in our current approach, we evolve our agents under isolated environmental pressures i.e., cases where agents are heavily biased to evolve to solve a single task. However, in the natural world, animals have evolved to jointly solve diverse tasks found in their ecological niches. While our framework can be easily extended for diverse visual tasks, isolated scenarios help us understand the extreme cases.

Our results point toward open technical challenges that will enable a wider variety of hypothesis to be tested. For instance, beyond single-task settings, multitask environments (e.g., Detection+Tracking) can impose conflicting demands, potentially revealing new trade-offs absent in single-task settings. Our framework can be extended to compose visual task objectives that test these trade-offs. In addition, multiagent vision coevolution, such as predator-prey or cooperative foraging, can also be simulated and studied. On the simulation side, incorporating richer light properties like spectral, polarization, or temporal sampling can also extend this to work to animals that see beyond visible light. In addition, future work could include incorporating biophysical models of vision (*36*) or replace neural networks with mechanistic circuits derived from fly connectomes (*11*, *78*).

Our framework provides a discovery tool by enabling large-scale computational evolution of vision in embodied artificial agents. For biologists and cognitive scientists, this approach allows systematic manipulation of key variables to test alternative hypothesis or counterfactuals, such as isolating the effects of optical elements from neural processing or testing how specific environmental pressures drive eye morphology. Much like natural and artificial evolution (*79*), our framework also demonstrates remarkable creativity in discovering solutions, for instance, it independently evolved distributed eyelet-type architectures without being explicitly designed to do so. For engineers, these evolutionary simulations reveal design principles for artificial vision systems, particularly valuable when optimizing for practical constraints like energy efficiency and manufacturability (*13*, *80*, *81*).

## MATERIALS AND METHODS

### Learning loop

The learning loop is the mechanism by which we score each agent. Via reinforcement learning, we train the brain of the agent (i.e., neural network parameters). Reinforcement learning serves as a mechanism for learning representations of the environment through interactions with it. The subsequent score, or fitness, of the agent is determined from the average reward it receives over six evaluation episodes after training. We use an open-source implementation of the PPO algorithm (*52*, *82*). Each agent is trained up to 1 million total steps, although training may be terminated early if no improvement is found after five evaluations.

Reinforcement learning algorithms have been shown to have a strong dependence on the random seed used to initialize the environment (*83*). Thus, during the evolution loop, we allow the same agent genotype to be sampled multiple times. In addition, each agent’s training loop is initialized with a unique random seed such that configurations sampled with the same genotype are not subject to the same seed. This allows for a more robust evaluation of the agent’s performance.

### Observations

An agent interacts with its environment through actions based on its observations. The observations are created by compositing the images captured from each eye. For example, if an agent’s vision system consists of five eyes with four photoreceptors in each eye, the resulting observation by the full agent “eye” at each time step will be a tensor of size 5 by 4 by 1 by 3. Furthermore, for each eye, the previous observations are stacked in a memory buffer. If the memory buffer is of size 10, then a tensor of size 10 by 5 by 4 by 1 by 3 is provided to the underlying agent network. In addition to the visual stimuli, we provide a single boolean that describes whether an agent is in physical contact with an object at the current time step and the previous action that was taken. Although not needed for the agent to solve the tasks, we have found that providing contact information and previous action as observations led to convergence nearly twice as fast; in an evolutionary search context, this speed-up significantly improves the overall optimization time.

### Reward function

The reward function is used in RL to drive policy optimization toward some desired observation and action mapping. In our case, each task has a unique reward function

$$R_{\text{NAVIGATION}}=\lambda \;\left(∥x_{t}-x_{0}∥-∥x_{t-1}-x_{0}∥\right)+w_{g}+w_{c}$$ (1)

$$R_{\text{DETECTION}}=\lambda \;\left(∥x_{t}-x_{f}∥-∥x_{t-1}-x_{f}∥\right)+w_{g}+w_{a}+w_{c}$$ (2)

$$R_{\text{TRACKING}}=\lambda \;\left(∥x_{t}-x_{f}∥-∥x_{t-1}-x_{f}∥\right)+w_{g}+w_{a}+w_{c}$$ (3)

where $R_{X}$ is the reward at time *t* for each task, λ is a scaling factor, $x_{t}$ and $x_{t-1}$ are the position of the agent at time t and t−1, respectively, $x_{0}$ is the initial position of the agent, and $x_{f}$ is the position of the goal (i.e., end of maze for Navigation and goal object in Detection). The w variables are nonzero when certain conditions are met. $w_{g}$ and $w_{a}$ indicate the reward/penalty given for reaching the goal and adversary, respectively. $w_{c}$ is the penalty for contacting a wall. In essence, in the Navigation task, the agent is incentivized to move from its initial position as fast as possible. In the Detection and Tracking tasks, the agent is incentivized to navigate to the goal as quickly as it can. During training, λ = 0.25, $w_{g}=1$, $w_{a}=-1$, and $w_{c}=-1$. In addition, when an agent reaches the goal or adversary, the episode terminates.

### Fitness function

As compared to the reward function, the fitness function is used to evaluate the current performance of an agent. Where the reward function is used to inform the RL algorithm for its weight optimization, the fitness function informs the evolutionary search algorithm for further selection and mutation. For each task, the fitness function $F_{X}$ in generation g is identical to $R_{X}$, except with different weights to emphasize the relative performance difference between agents. During fitness evaluation, λ = 1.5, $w_{g}=10$, $w_{a}=-10$, and $w_{c}=-2$. Instead of terminating when the goal or adversary is reached, in evaluation, the object is respawned, and the agent continues to solve the task.

### Evolution loop

Evolving the full agent visual genotype, with a total of >∼10^20^ possible combinations, necessitates “intelligent” optimization. The vast size of the search space alone means all combinations cannot be tested in a timely fashion. Strategically selecting morphologies that simultaneously explore new configurations and exploit previously gained knowledge is imperative to not waste resources on suboptimal solutions. We accomplish this intelligent search mechanism through the integration of ES (*48*). ES is a broad optimization technique that is inspired by natural evolution and operates by iteratively refining a population of candidate solutions through processes such as mutation, selection, and adaptation. Unlike traditional genetic algorithms, ES emphasizes mutation over crossover and is particularly well suited for optimizing continuous, high-dimensional spaces.

We use a population size of 16 agents and evolve for 50 to 100 generations depending on the experiment. The specific ES algorithm we use is the CMA-ES (*84*). CMA-ES is a variant of ES that adapts the mutation distribution based on the covariance matrix of the population. This adaptation allows for faster convergence and better exploration of the search space. We use the open-source implementation of CMA-ES provided by nevergrad (*51*). Hyperparameters for CMA-ES can be found in the Supplementary Materials.

### Robustness

We ensure robustness in our experiments by explicitly setting the random seeds at two levels: one at the evolutionary search level (outer loop) and one at the RL. This design choice isolates evolutionary stochasticity from reinforcement learning variability, allowing us to specifically test whether different evolutionary search trajectories discover similar optimal solutions.

### Intrarun robustness

For a specific evolutionary run, we set a random seed, which remains fixed for the duration of the experiment. This seed only affects the random choices associated with evolutionary selection and is set to the random number generator attached to the selection mechanism in nevergrad (*51*). Subsequently, the seed assigned to the RL experiment is calculated based on the agent’s unique identifier according to the following formula

$${\text{seed}}_{\text{RL}}=\text{num}\_\text{envs}\times \left(\text{generation}\times \text{population\_size}+\text{rank}\right)+\text{agent\_id}$$ (4)

Thus, each agent will be evaluated with a unique random seed. In addition, we have configured the evolutionary search algorithm (CMA-ES) to (i) re-evaluate the best performing agent from the previous population (elitist) and (ii) allow morphologies to be selected more than once. Using Eq. 4 allows us to evaluate the same morphologies multiple times with different random seeds, leading to a more robust evaluation metric that is less susceptible to noise from individual RL experiments. This mechanism, along with evaluating upward of ~3200 independent agent morphologies, helps ensure robustness to lucky random seeds.

### Inter-run robustness

To evaluate robustness between evolutionary runs, we conducted five total evolutionary experiments with different CMA-ES seeds. We fix Eq. 4 but initialize the evolutionary search algorithm with a different seed. This leads to a unique initial population and, thus, a new experiment with different conditions. We rerun each experiment five times using the following seeds: 0, 42, 420, 1337, and 4002. The experimental results can be seen in Fig. 3 and fig. S1.

### Agent phenotype

An agent’s phenotype is the physical manifestation of its genotype. The phenotype is the realized form that interacts within the environment that acquires and acts on observed stimuli. An agent in this work is represented as a fixed radius sphere with eyes facing outward and distributed uniformly along its equator. The agent is embodied and can control its direction and speed using its underlying policy, which are used to actuate the joints to move the agent in the simulation environment. Our framework also allows for more complex dynamical systems, and controllers could be used as well. Last, in the case of the Tracking task, we assign computed action profiles to the goal and adversary to move to random locations within the environment.

### Agent’s genotype

The genotype encodes the instructions to create the agent’s eye and cognitive system that learns task behavior. The vision genotype is further divided into three clusters that are mutated independently and incorporate both continuous and discrete parameters. The clusters are morphological, optical, and neural (Fig. 2). Rather than modeling complex biological mechanisms like photoreceptor dynamics, we implement these genes to encode capture of light from a physics-based rendering perspective. We believe that this model captures the essential functional properties needed to evolve vision while remaining computationally tractable in modern embodied simulators. Our full encoding scheme can represent ~10^20^ unique agent vision types.

### Morphological genes

The morphological genes define properties used to spatially sample the environment, such as the number of eyes, their placement (determined by placement range), and FOV. We model the agent as a sphere of fixed radius of 0.2 units with eyes distributed uniformly along its equator. Thus, the placement of each eye is also dependent on both the number of eyes, and a placement range (i.e., the maximum angle from latitude 0°) that eyes are uniformly distributed within. For instance, if an agent has three eyes and the placement range is 90°, the eyes are placed at −45°, 0°, and 45°; however, a placement range of 20° will result in three forward-facing eyes at −10°, 0°, and 10°. The orientation of each eye is determined by the normal vector from the agent’s body. We assume bilateral symmetry, consistent with the observation that most animals have bilateral symmetry (*85*). The FOV is a continuous value between 1°, 100°]. We visualize the effect of sampling different morphological genes on agent’s vision in figs. S7 to S9.

### Optical genes

**Fig. 6.**
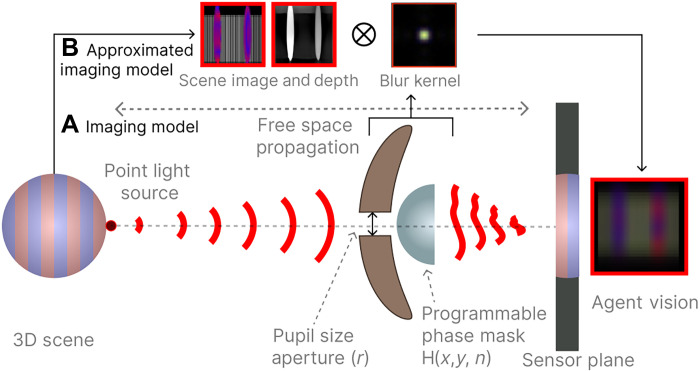
Imaging model. Our simulation implements both wave and geometric optics using OpenGL (*90*). (**A**) Our scene imaging model shows how a depth-dependent blur kernel is derived: Light from a point source in the 3D scene propagates through free space, passing through a pupil plane, which is composed of (i) an aperture with a variable aperture radius (*r*) and (ii) a programmable phase mask with height map H(x,y) and refractive index n, before forming an image on the sensor plane. (**B**) The approximated model uses a 2D convolution between the scene, a depth map, and a single blur kernel using the farfield approximation (i.e., the PSF) for computational efficiency.

The optical genes describe how each eye interacts with incoming light in a physically plausible way. It encompasses a programmable DOE, or a phase mask, that modulates the phase of the incoming light (represented as a 4 by 4 array with values in [0, 1]), refractive index (η∈[1.0,2.0]) and pupil radius, or aperture a∈[0,1] that is scaled dynamically with sensor size as r=a×L, where L is the sensor size. Note that pupil size of a=1 results in an “open eye” as the pupil and sensor size are equal. We use continuous parameters for phase mask, pupil radius, and refractive index and then upsample the phase mask to a size of 51, 51 to generate smoother PSFs. The optical gene can also be disabled, in which case a rasterization-based imaging model is used to create visual stimuli for the agent; this model is analogous to an eye with a perfect lens, i.e., no blur. Our framework does not limit the size of the phase mask; however, we choose 4 by 4 to make the problem computationally tractable. We visualize the effect of sampling different optical genes on agent’s vision in figs. S10 and S11 and Fig. 6.

### Neural genes

The neural genes determine the learning capacity of the agents by encoding properties of the agent’s neural network, such as temporal memory size and number of neurons. The memory size represents the number of historical frames relative to the current frame the agent has access to; for instance, a memory size of five means the agent’s visual stimulus is a flattened vector each composed of the current frame and the previous five frames. For “What if the brain became larger?”, we sample different neural genes from an underlying neural network architecture that has two identically sized hidden layers. The number of neurons in the hidden layers is an integer mutation parameter that can be between 1 and 512. Although we do not present experiments in this work evolving the neural network architecture, it is possible to mutate the underlying architecture (i.e., add layers, change activation functions, etc.) in our framework.

### Mutation operators

The agent’s genotype is designed with specific mutation operators for each parameter type. For continuous parameters (such as FOV, phase mask values, pupil radius scaling factor, and refractive index), mutations occur through Gaussian perturbation within their defined ranges. For discrete integer parameters (such as number of eyes and number of neurons), mutations increment or decrement the current value while respecting the parameter bounds. Bilateral symmetry is preserved during all morphological mutations.

### Experimental control

In each experiment, we enable specific mutations to isolate and study specific aspects of vision evolution. This controlled approach allows us to systematically investigate the evolution of different visual traits.

### Imaging model

To produce environmental pressures based on physics of light transport, we use a physically based imaging model for the agent’s eye. This ensures that agents must evolve their optical genes to contend with physical laws that limit visual systems. Thus, our imaging model consists of a programmable lens described by a phase mask, refractive index, and amplitude modulating (aperture) elements described by a pupil/aperture radius. The agent’s retina is emulated by a discretized pixel at the sensor plane at a focal length distance away from the pupil (Fig. 6).

All imaging systems capture the scene as an optically encoded image on to the sensor plane. These optical encodings are commonly referred to as the blur or PSF and are dependent on the phase and amplitude of the pupil function along with wavelength and depth of the scene point. We follow the wave propagation model described in (*86*–*88*) to estimate the PSF. Since this model will be applied per eye per step of the agent, there is an inherent trade-off between speed of rendering and accuracy of the imaging model. Thus, we assume depth-independent PSFs to limit the number of depth-convolutions, to one convolution, per eye. In addition, to reduce the number of evolvable parameters for the amplitude mask, we also assume a circular amplitude mask parametrized by a single value (pupil radius). Our framework is not limited to these assumptions.

Given a point light source at a distance z and the pupil function $P\left(x,y\right)=A\left(x,y\right)\text{exp}\left[i\times t_{\phi }\left(x,y\right)\right]$ (Fig. 6), the response of the agent’s eye can be measure by the PSF. The PSF at the sensor plane s distance away from the pupil plane is described as

$$\text{PS}F_{\lambda {,}_{z}}\left(x^{\prime },y^{\prime }\right)={\left|F^{-1}\left\{F\left[P\left(x,y\right)U_{\text{in}}\left(x,y\right)\right]H_{s}\left(f_{x},f_{y}\right)\right\}\right|}^{2}$$ (5)

where $H_{s}\left(\cdot \right)$ represents the field propagation transfer function (*89*) for distance s with $\left(f_{x},f_{y}\right)$ as the spatial frequencies given as

$$H_{s}\left(f_{x},f_{y}\right)=\text{exp}\left[\mathrm{iks}\sqrt{1-{\left(\lambda f_{x}\right)}^{2}-{\left(\lambda f_{y}\right)}^{2}}\right]$$ (6)

where $k=\frac{2\pi }{\lambda }$ is the wave number; $U_{\text{in}}\left(x,y\right)$ denotes the complex-valued wave field immediately before the lens, which for a point light source is given as

$$U_{\text{in}}\left(x,y\right)=\text{exp}\left(\mathrm{ik}\sqrt{x^{2}+y^{2}+z^{2}}\right)$$ (7)

F{·} is the 2D Fourier transform, $\left(x^{\prime },y^{\prime }\right)$ are the spatial coordinates on the camera plane, and (x,y) are the coordinates on the lens plane.

The pupil function $P\left(x,y\right)=A\text{exp}\left[i\times t_{\phi }\left(x,y\right)\right]$ contains an amplitude modulate function A(x,y) and a phase modulation function $t_{\phi }\left(x,y\right)$. To keep the number of evolvable parameters small, we assume a circular amplitude mask: ${\left(x-\frac{W}{2}\right)}^{2}+{\left(y-\frac{H}{2}\right)}^{2}=r^{2}$ where r∈[0,1] and is a mutated parameter. The phase modulation function is represented by $t_{\phi }\left(x,y\right)=e^{i\frac{2\pi }{\lambda }\phi \left(x,y\right)}$ in Eq. 5 is generated by the lens surface profile ϕ(x,y), which in our case is a mutating square 2D phase-mask array of size 4 by 4, where ϕ(x,y)∈[0,1]

$$U_{\text{in}}\left(x,y\right)=\text{exp}\left(\mathrm{ik}\sqrt{x^{2}+y^{2}+z^{2}}\right)$$ (8)

Last, our agent’s image formation follows a shift-invariant convolution of the image and the depth-independent PSF to yield the final image, $I_{\text{ℓ}}$, perceived by the agent

$$I_{\ell }=\left[S_{\ell }\left(H_{\ell }*X_{\mathrm{\ell ;}}\right)\times r^{2}\right]+N_{\ell }$$ (9)

where the subindex ℓ denotes the color channel; $X_{\ell }\in {\mathbb{R}}_{+}^{w\times h}$ represents the underlying scene with w×h pixels; $H_{\ell }$ represents the discretized version of the PSF in Eq. 5; $N_{\ell }\in {\mathbb{R}}^{w\times h}$ denotes the Gaussian noise in the sensor; $S_{\ell }\left(\cdot \right):{\mathbb{R}}^{w\times h}\to {\mathbb{R}}^{w\times h}$ is the camera response function, modeled as a linear operator; multiplication by *r*^2^ denotes changing light throughput onto the pixel area as a result of the aperture; and * denotes the 2D convolution operation. The light throughput falls quadratically because light falls onto a sensor plane, which has finite area in *x* and *y*.

In practice, the discretized version of the PSF [*H*, *W* in A(x,y) and $t_{\phi }\left(x,y\right)$] is of size (H+1,W+1) where (H,W) is the resolution of the agent’s eye. This is an explicit choice to make the PSF larger than the image to enable a full blur on the eye when the aperture is fully open. To make sure that angular scene resolution is maintained, the scene image $X_{\ell }$ is rendered by padding $I_{\ell }$ of size (H,W) to $\left[H+\left(2*\frac{H+1}{2}\right),W+\left(2*\frac{W+1}{2}\right)\right]$.

This ensures that like a real eye, the corner photoreceptors also accumulate light from outer regions directly due to the aperture size. This also means that closing the aperture also helps with reducing the total effective FOV of the agent’s eye and less blur, which is how agents control the blur in the phase I experiment in “What if eyes could bend light?”. For pinhole eyes, the FOV becomes equivalent to the encoded FOV in the agent’s morphological gene as the blur kernel is very small.

### Simulated environment

This framework is built on top of the MuJoCo physics engine (*33*) and within a gymnasium-style (*44*) setup. The underlying dynamics of each agent is governed by the MuJoCo physics, and images are rendered via a rasterization pipeline using the built-in OpenGL renderer. We customize this environment with the physically based rendering model.

In this work, we differentiate between a visual task and an environment. A visual task is the specific goal an agent is trying to achieve defined by the reward function. The environment is the physical space in which the agent is placed and so can contain multiple tasks. For instance, the same environment is used to train agents on both the Detection and tracking tasks; the only difference is the reward function and the movement of the goal/adversary. In addition to the physical positioning of objects in an environment, the textures, light, colors, etc. can be modified to create a diverse set of various environments.

Each environment also has walls, which can be organized as a boundary or in a maze-like configuration. These walls are rigid and contactable and provide barriers where the agent cannot escape. We can also add any textures on these walls. While we only train a single agent that has evolved and trained in an environment, we also have nontrainable agents. Nontrainable agents have a fixed action policy and have privileged information about the environment. For example, a nontrainable goal object in the Tracking task has a fixed policy that randomly samples actions to move in the environment.

## Supplementary Materials

### The PDF file includes:

Supplementary Text Figs. S1 to S11 Legends for movies S1 to S3 References
