# Supplementary material for: What if eye...? Computationally recreating vision evolution
Source: Sci Adv. 2025 Dec 17;11(51):eady2888. doi: 10.1126/sciadv.ady2888 (PMC12710701; doi:10.1126/sciadv.ady2888)
Supplement: Supplementary file 1 — Supplementary Text Figs. S1 to S11 Legends for movies S1 to S3 References [file sciadv.ady2888_sm.pdf]

Science Advances
AAAS

Supplementary Materials for

What if eye...? Computationally recreating vision evolution

Kushagra Tiwary *et al.*

Corresponding author: Kushagra Tiwary, ktiwary@mit.edu; Brian Cheung, cheungb@mit.edu

Sci. Adv. **11**, eady2888 (2025)
DOI: 10.1126/sciadv.ady2888

The PDF file includes:

Supplementary Text
Figs. S1 to S11
Legends for movies S1 to S3
References

Other Supplementary Material for this manuscript includes the following:

Movies S1 to S3

Supplementary Text

Analysis of Evolved Agents

Our analysis quantifies the optical performance of evolved vision systems using three metrics. The Point Spread Function (PSF) represents the system's response to a point source of light - how a perfect point gets "spread out" by the optical system. In 7, high-performing agents develop compact, symmetric PSFs indicating precise light focusing, while poor performers show diffuse, irregular patterns suggesting inefficient light management. A perfect PSF would appear as an infinitesimally small point, while real optical systems produce some degree of spread due to diffraction and optical imperfections.

MTF for Spatial Precision Analysis The Modulation Transfer Function (MTF), mathematically derived as the Fourier transform of the PSF, quantifies how well different spatial frequencies are preserved by the optical system. On the MTF plots in 7, the y-axis represents contrast preservation (from 0 to 1) while the x-axis shows spatial frequency in cycles/mm. The area above the noise floor ($\sim 10^{-2}$) represents useful spatial information — frequencies where the signal can be reliably distinguished from noise. Early-generation agents show erratic MTF curves with sharp dips below this noise floor, while later-generation agents maintain smooth curves above the noise floor up to 40 cycles/mm.

Image Quality Metric. These observations led us to develop an Image Quality metric that multiplies two factors: (1) the area under the MTF curve above the noise floor (marked in 7 by the dashed noise floor line), representing spatial precision, and (2) light throughput, which decreases quadratically with pupil radius. This metric captures the trade-off between spatial precision and light collection. While pinhole eyes can achieve good MTF performance, their limited light throughput constrains their overall image quality. Lens-based eyes resolve this trade-off by maintaining strong MTF performance while allowing larger apertures for better light collection.

Quantifying Signal-to-Noise Ratio in Evolved Agents. The PSNR and SSIM curves in 8 reveal limitations of these conventional metrics. Initially, with fully open apertures, both metrics show high values because extreme blur acts as a noise-reducing low-pass filter. However, this blurred vision makes the DETECTION task impossible, resulting in low agent fitness. As apertures begin to close (forming pinhole eyes), PSNR and SSIM decrease as the system preserves more high-frequency information but with increased noise due to limited light collection. When lensing is enabled at generation 30, we observe steady improvement in both metrics as the system evolves the ability to maintain high-frequency detail while collecting sufficient light.

Analyzing fitness trajectories for DETECTION task. In the DETECTION Task a fitness of $>$ 15 means that the agent has correctly detected food at least once (each food thas a $+10$ fitness score), and a fitness of 25 means that this has happened at least twice. In 8, we plot the median fitness for phase I which saturates at a fitness score of 10 which results in the agent not detecting any food. However, median fitness for Phase II by generation 130 is $>$ 18 which shows that

evolution has discovered a reliable way to continuously detect food from 2 poison objects. Moreover, the best agents can do it multiple times as the max fitness score for Phase II is > 25. Comparatively, agents with max fitness for Phase I (solid purple line) detect food at least once which is mostly a result of randomness and getting lucky.

Randomness in Agent Behavior. Notably, some agents with excellent optical properties (high MTF and light throughput) show slightly lower rewards due to the stochastic nature of reinforcement learning. This variance in reward despite similar optical quality suggests that the relationship between optical performance and task success is not purely deterministic - better vision enables but does not guarantee better task performance.

$$\text{CPD} = \frac{1}{2 \cdot \min\left(\frac{\text{lon\_range}}{\text{num\_eyes} - 1}, \frac{\text{fov}}{\text{resolution}}\right)} \quad (9)$$

$$\text{FOV} = 2\arctan\left(\frac{\text{sensor\_size}}{2 \times \text{focal\_length}}\right) \quad (10)$$

$$\text{lon\_range} = \text{sensor\_size} \times \text{num\_eyes} \quad (11)$$

Quantifying Agent Vision through CPD. We also quantify an agent's morphological and optical genotype in cycles per degree (CPD). The cycles per degree is a measure of the spatial frequency observable to the imaging system; a higher CPD value corresponds to a better ability to resolve fine spatial details and distinguish closely spaced features in the visual scene (*32*). CPD is also a commonly used metric to measure visual capabilities in real-life animals (*32*). Eq. 9, Eq. 10, and Eq. 11 are used to calculate CPD for our agents.

Genotype and its relation to the Plenoptic Design Space

The vision genotype of the agent can be understood as operating on the Plenoptic function, which describes the complete flow of light in a scene (*91*). We can conceptually think about biological vision evolution as directly evolving to capture different dimensions of the Plenoptic function. Our current implementation demonstrates that computational evolution can also sample from the Plenoptic function (*91*): we allow evolution to explore a subset of Plenoptic dimensions such as placement, orientation, optical constraints, movement of the agent etc. We believe this framework can naturally extend to encompass the full Plenoptic representation of light - including spectral sensitivity, polarization detection, and varied spatiotemporal resolutions. Just as our computational experiments have shown evolution discovering diverse and creative solutions within a limited set of visual parameters, expanding the genotype to sample from the complete Plenoptic dimensions would enable the discovery of even more sophisticated visual systems, analogous to those found in nature. For instance, the mantis shrimp (stomatopods) evolved 16 different photoreceptor types that can detect both linear and circular polarized light (*92*), while jumping spiders (Salticidae: Dendryphantinae) developed a unique combination that provide both high acuity and wide-field motion detection (*93*). Our genotype enables co-evolution of eyes, neural circuitry and subsequent behavior (learned through reinforcement learning) and provides a unified way to think about vision evolution as a creative optimization process operating directly on the fundamental properties of light. As we expand the genotype of the available Plenoptic dimensions, we expect to see the emergence of increasingly sophisticated

and novel visual systems that may parallel, or even exceed, the remarkable diversity found in biological evolution.

Acuity-Neural Processing Trade-offs and Task-Specific Scaling

In our framework, we systematically explore how visual task performance emerges from the interplay of three key components. The first component is the eye's physical characteristics, measured in cycles per degree (CPD), which determines the ability to resolve spatial detail. The second is neural capacity, where we vary the number of parameters in the vision-processing layers.

Our parameter sweep reveals emergent power law scaling relationships between sensory acuity and neural capacity 11. The relative fitness plots (top row) demonstrate that NAVIGATION achieves high performance (>0.8) at lower CPDs (~0.05) with modest neural capacity (~8000 parameters). DETECTION and TRACKING tasks show a distinct scaling pattern, requiring both higher CPDs (>0.3) and larger networks (>40,000 parameters) for comparable fitness levels. The error plots (bottom row) reveal fundamental constraints in how these capabilities emerge. At fixed CPD values, increasing neural capacity follows characteristic power law improvements until hitting task-specific performance ceilings. These ceilings are particularly evident in the scattered error distributions, where higher CPDs enable lower minimum error rates across all tasks. This demonstrates that poor visual acuity creates a fundamental bottleneck that cannot be overcome by simply scaling neural capacity. Notably, DETECTION and TRACKING display continuous improvements in error rates as both CPD and network size increase, suggesting these tasks benefit from simultaneous scaling of both sensory and neural resources.
These computational scaling relationships emerged spontaneously through evolution in our framework, revealing how physical constraints in sensory acuity interact with neural processing capacity to shape task performance. The distinct scaling patterns across tasks, particularly the earlier saturation in NAVIGATION compared to DETECTION and TRACKING, suggest a natural hierarchy in the visual processing demands of different behaviors (*28*). This emergent relationship between sensory hardware and neural processing mirrors both biological evolution (*94*, *95*) and contemporary artificial intelligence scaling laws (*30*, *74*, *96*).
For temporal performance, we find that performance saturates beyond 10 frames across all configurations. This is particularly evident in the TRACKING task, where agents are incentivized to complete objectives quickly, typically achieving success in under 10 frames. This reveals an optimal balance between temporal information and computational efficiency that varies by task complexity.

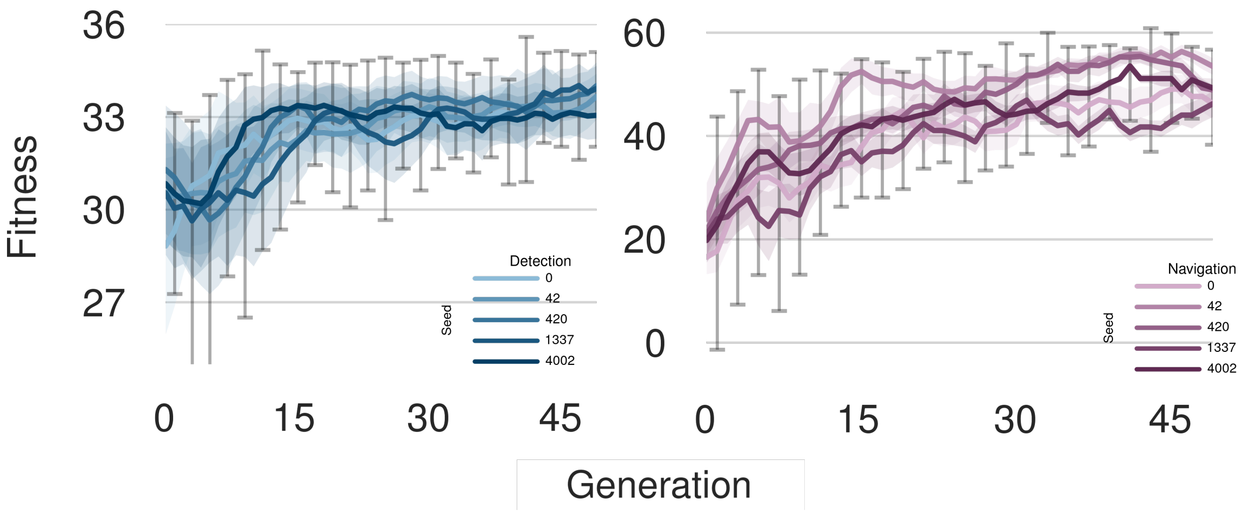

Fig. S1: Inter-experiment robustness evaluation: *We ran repeated full evolutionary experiments with different initial random seeds to verify the results are robust to initial conditions. We evaluate the DETECTION and NAVIGATION environments using 5 different seeds each: 0, 42, 420, 1337, 4002. These plots show the mean fitness for each experiment as a dark line and the 95% confidence interval for all experiments at each generation. These results demonstrate that our framework, and the results we present herein, are reproducible and robust.*

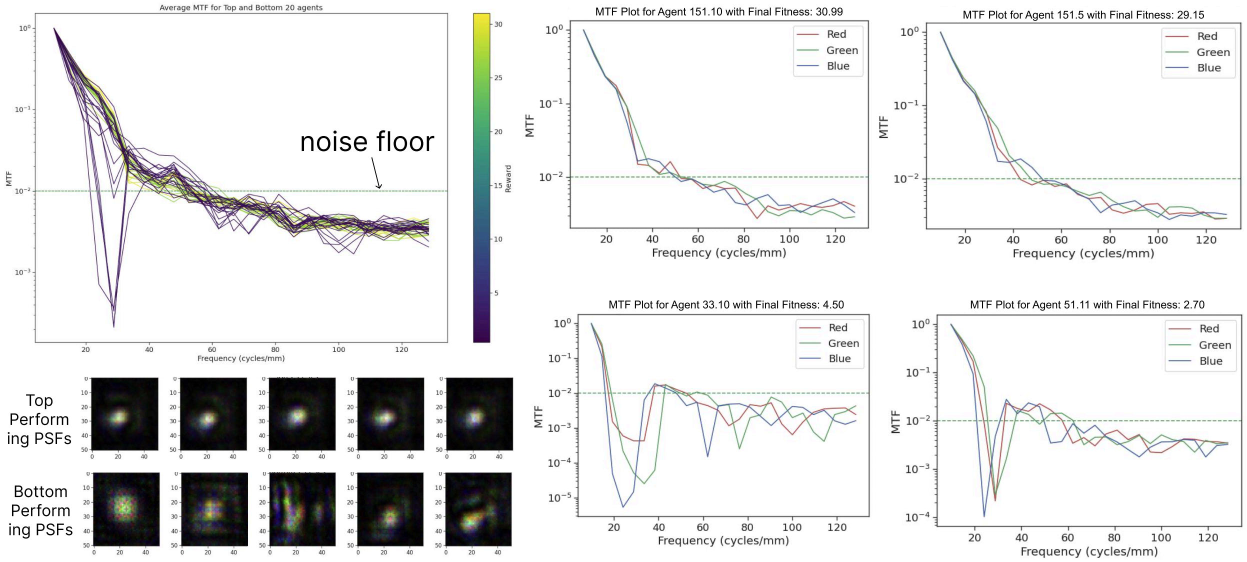

Fig. S2: Modulation Transfer Function (MTF) analysis of evolved vision systems: *We analyze the spatial frequency response of evolved vision systems using MTF curves, which quantify how well different spatial frequencies are preserved. Left: Average MTF curves for top 25 and bottom 25 performing agents, colored by reward. The horizontal dashed line indicates the noise floor, below which spatial information becomes unreliable. Bottom: Point Spread Functions (PSFs) for the best and worst performing agents, showing the characteristic light distribution patterns. Top Performing Agents develop compact and symmetric PSFs even though we don't enforce any symmetry in our setup. Right: Individual MTF curves for agents at different evolutionary stages (Generation 33, 51, and 151) and performance levels. Early generations (Gen 32, 51) show erratic frequency responses with significant dips, indicating poor optical performance. By Generation 151, high-performing agents develop smooth MTF curves that maintain good contrast above the noise floor up to 40 cycles/mm, demonstrating evolution of effective lens-based vision systems. The RGB channels show similar responses, suggesting achromatic optimization of the optical system. Note that the dispersion in the PSFs will be clearer in the pdf version compared to the print.*

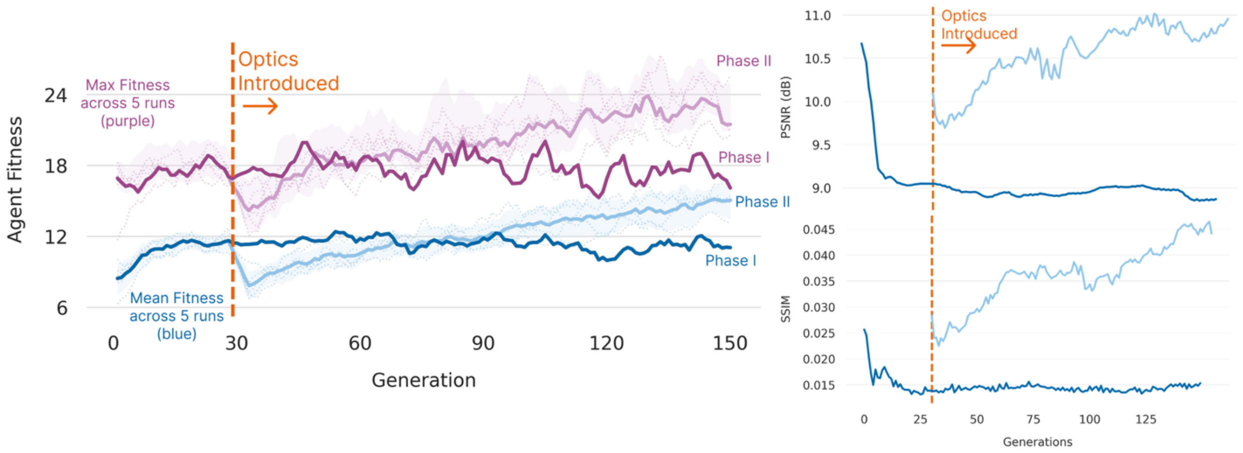

Fig. S3: Agent fitness, PSNR (dB) and SSIM over generations: *We show additional graphs from Section 2.4 that show steady increase in agent fitness. The main plot tracks both maximum (purple) and mean (blue) agent fitness across 5 independent evolution runs for Phase I (solid) and Phase II (opaque). In Phase I, median fitness saturates at 10 indicating no food detection, while in Phase II, median fitness exceeds 18 by generation 130, demonstrating evolved capability for reliable food detection. The maximum fitness of >25 in Phase II indicates multiple successful detections, whereas Phase I's maximum fitness shows only occasional random successes. To compute SSIM and PSNR we render a rastered version of the image using the pinhole camera model as the reference image. The scene rendered as a raster image is shown in Fig. S8. For PSNR we show a substantial increase from the pinhole eyes. This shows that lensing significantly improves the signal-to-noise tradeoff that we discussed in Section 2.4. We can also compare the SSIM between Phase I and II. Unlike PSNR, SSIM is a perception-based model that considers changes in structural information between reference and target images. The SSIM increases are small as it relies on pixel wise calculations, but the trend demonstrates that lens-based eyes better preserve image structure while maintaining higher light collection compared to pinhole eyes, enabling more reliable discrimination between similar visual features.*

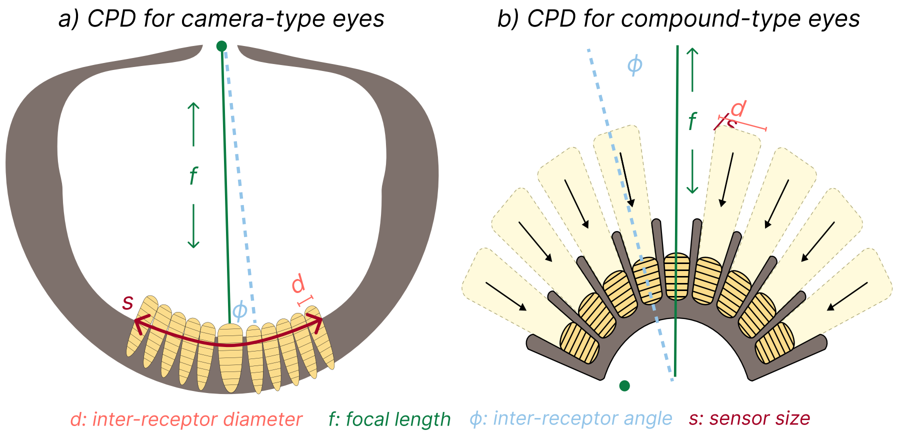

Fig. S4: Comparative analysis of cycles per degree (CPD) calculations for agents with different eye morphologies. *We show the schematic representation of two distinct eye architectures and their corresponding CPD calculation methods. (a) An example camera-type eye configuration is shown with a single eye with 11 photoreceptors, characterized by focal length (f), inter-receptor diameter (d), inter-receptor angle (ϕ), and sensor size (s). (b) An example Compound-type eye configuration with 9 individual eyes, each containing a single photoreceptor. The field of view (FOV) is determined by the relationship between sensor size and focal length, while the range of placement of eyes denoted as longitudinal range, lon_range, is calculated as the product of sensor size and number of eyes. For our agents, we compute CPD using the minimum ratio of longitudinal range to the number of eyes minus one, and the field of view to resolution ratio (FOV/resolution). Note that if that the compound eye had more than one photoreceptor d would be a per-photoreceptor measurement and s would remain the same, however, in this configuration they are the same since we are showing a simpler case of one photoreceptor per eye.*

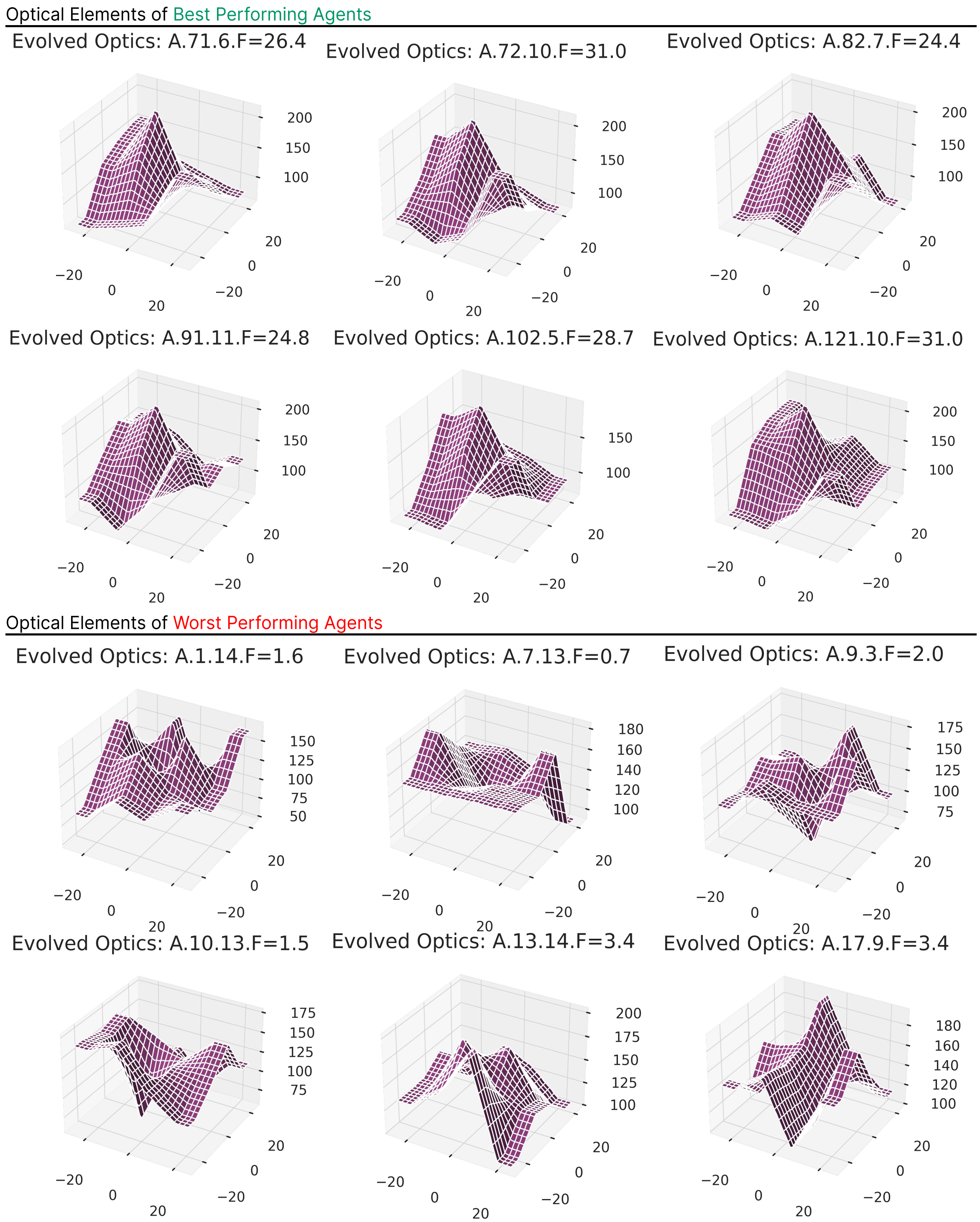

Fig. S5: Comparison of evolved optical elements between best and worst performing agents. *Three-dimensional surface plots showing the optical response patterns for the top six (upper panel) and bottom six (lower panel) performing agents. Best performing agents (F=24.4-31.0) exhibit well-defined, singular peak formations with smooth gradients, while worst performing agents (F=0.7-3.4) display irregular, multi-peaked patterns with abrupt transitions. Each plot represents a unique evolved optical configuration denoted by its agent identifier (A) and corresponding fitness score (F).*

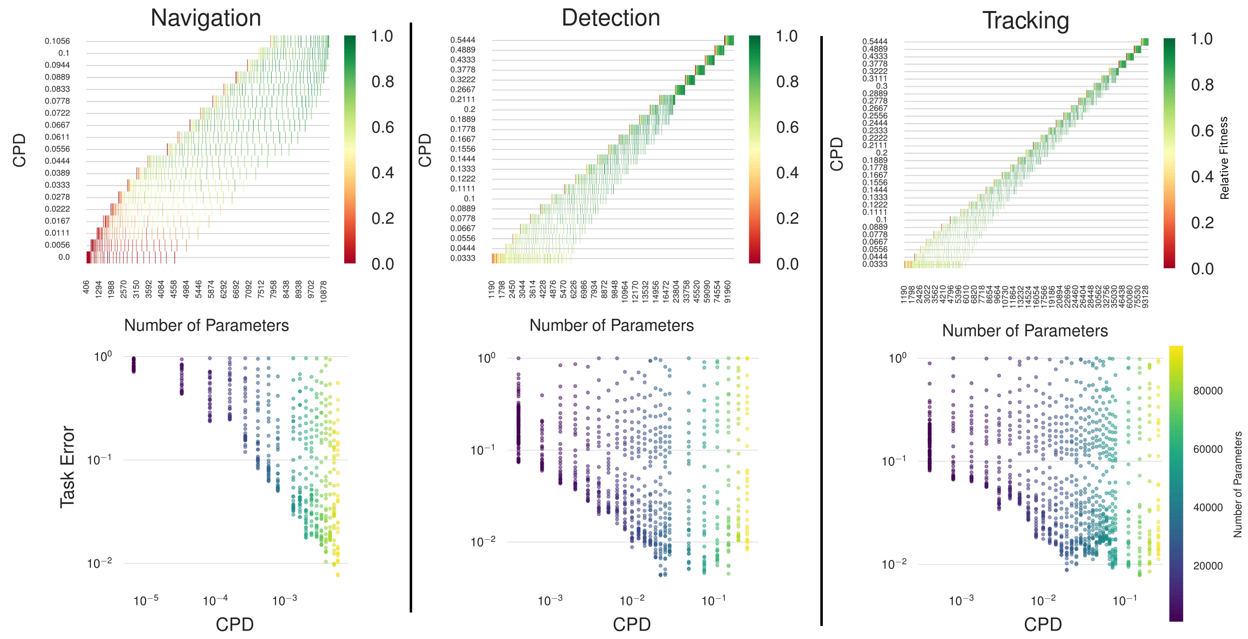

Fig. S6: Dense parameter analysis revealing task-specific relationships between sensory acuity and neural processing. Top: *Performance visualization showing individual trials (vertical lines) across CPD values and network sizes for* NAVIGATION *(left), detection (middle), and tracking (right).* ***Bottom:*** *Corresponding scatter plots with log-scaled axes demonstrate how error rates vary with CPD for different network sizes (indicated by color intensity). The distinct patterns across tasks support our findings about task-dependent scaling relationships*

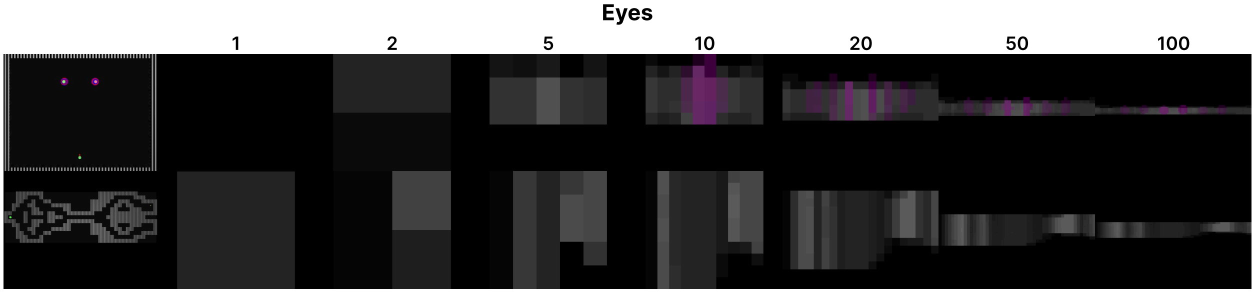

Fig. S7: Sampling greater number of eyes by modifications to the morphological gene: *We display our agent's vision captured with progressively more number of eyes. Top row shows progressively increasing eyes allows for larger FOV of the scene and creates multiple copies of the spheres from slightly different perspectives in each eye. The bottom row shows that for the* NAVIGATION *task number of eyes allows the agent to see different parts of the wall which it uses to orient itself against wall collisions.*

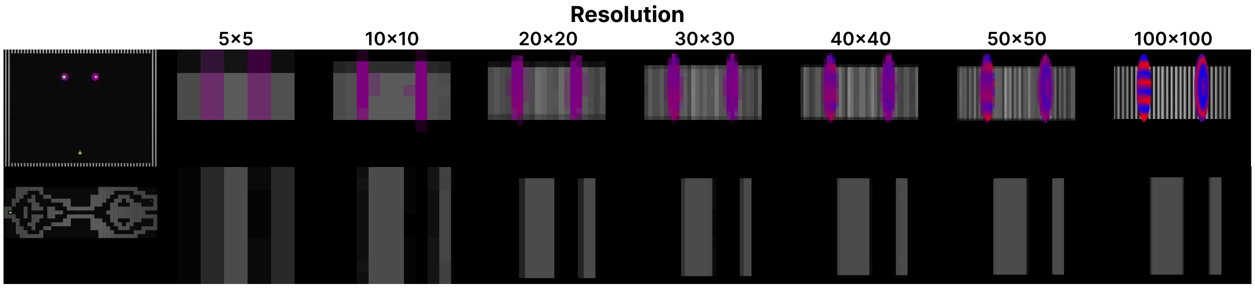

Fig. S8: Sampling larger resolutions by modifications to the optical gene: *We display our agent's vision captured with progressively larger resolutions. Top row shows progressively increasing resolution resolved the difference between food and poison which can be differentiated with the orientation of the stripes. The bottom row shows that for the* NAVIGATION *task resolution helps resolve the stripes on the wall.*

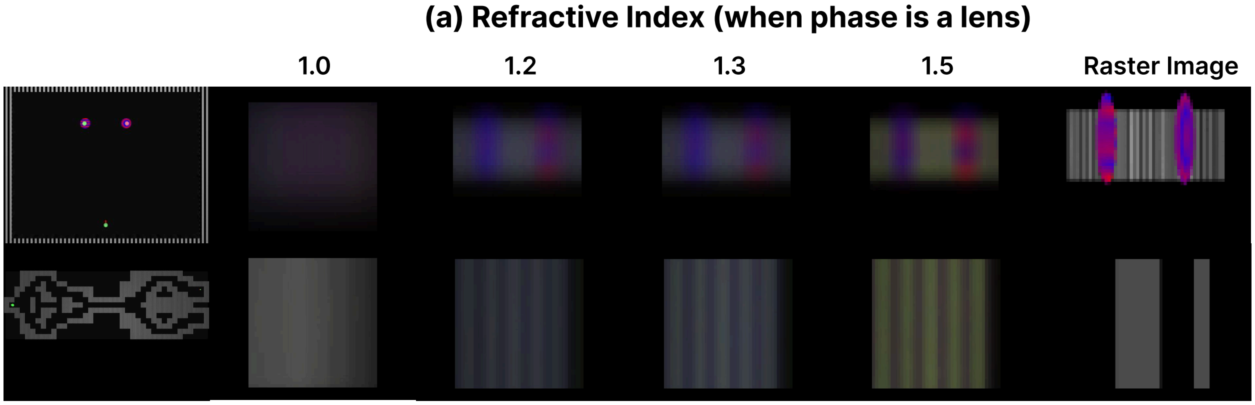

Fig. S9: Sampling refractive indices in the optical gene: *We illustrate examples of sampled refractive indices within the optical gene for* DETECTION *(top row) and* NAVIGATION *(bottom row) tasks. For a fixed phase mask (a perfect lens) increases in refractive index* produces sharper images.

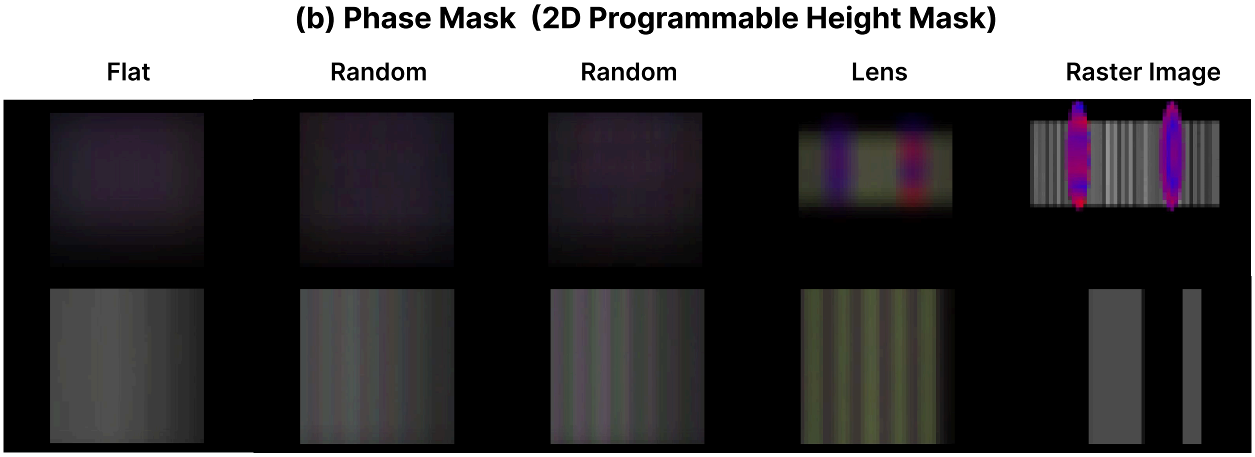

Fig. S10: Sampling optical elements in the optical gene: *We illustrate examples of sampled optical elements (phase masks) within the optical gene for* DETECTION *(top row) and* NAVIGATION *(bottom row) tasks. The figure shows flat, and two randomly samples phase masks which shows the complexity of the design space. These visualizations also demonstrate that while using a lens is a major innovation in eye design, creating a focused lens is a hard problem that evolution solved well.*

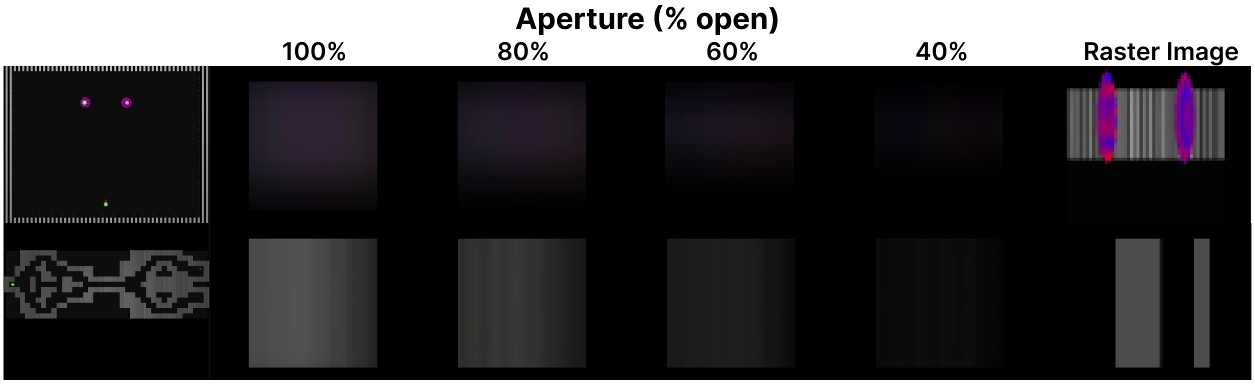

Fig. S11: Sampling different apertures by modifying the optical gene: *We display our agent's vision captured with progressively smaller apertures, demonstrating how reducing the aperture size leads to increased image sharpness. However, as the aperture closes, the signal strength decreases quadratically with its radius, leading to higher noise levels. The balance between sharpness and noise is a critical factor for agents to successfully complete their visuomotor tasks.*

Movie. S1: *Emergence of forward-facing camera-type eyes when agents evolve for the DETECTION Task.*

Movie. S2: *Emergence of compound and finally eyelet eyes when agents evolve for the NAVIGATION Task.*

Movie. S3: *Evolution of lens-based eyes from open eyes.*

REFERENCES

1. M. Land, D.-E. Nilsson, *Animal Eyes* (Oxford Univ. Press, 2002).

2. R. D. Fernald, Casting a genetic light on the evolution of eyes. *Science* **313**, 1914–1918 (2006).

3. T. W. Cronin, S. Johnsen, N. J. Marshall, E. J. Warrant, *Visual Ecology* (Princeton Univ. Press, 2014).

4. J. Pearl, D. Mackenzie, *The Book of Why: The New Science of Cause and Effect* (Basic Books, 2018).

5. W. G. Walter, A machine that learns. *Sci. Am.* **185**, 60–63 (1951).

6. O. Holland, The first biologically inspired robots. *Robotica* **21**, 351–363 (2003).

7. S. Nolfi, J. Bongard, P. Husbands, D. Floreano, *Evolutionary Robotics* (Springer International Publishing, 2016); https://doi.org/10.1007/978-3-319-32552-1_76.

8. J. C. Bongard, Evolutionary robotics. *Commun. ACM* **56**, 74–83 (2013).

9. J. Krause, A. F. Winfield, J.-L. Deneubourg, Interactive robots in experimental biology. *Trends Ecol. Evol.* **26**, 369–375 (2011).

10. V. Trianni, Evolutionary robotics: Model or design? *Front. Robot. AI* **1**, 13 (2014).

11. J. K. Lappalainen, F. D. Tschopp, S. Prakhya, M. McGill, A. Nern, K. Shinomiya, S. Takemura, E. Gruntman, J. H. Macke, S. C. Turaga, Connectome-constrained networks predict neural activity across the fly visual system. *Nature* **634**, 1132–1140 (2024).

12. D. Floreano, S. Nolfi, "Adaptive behavior in competing co-evolving species" in *4th European Conference on Artificial Life* (1997), pp. 378–387.

13. H. Lipson, J. B. Pollack, Automatic design and manufacture of robotic lifeforms. *Nature* **406**, 974–978 (2000).

14. K. Miras, E. Ferrante, A. E. Eiben, Environmental influences on evolvable robots. *PLOS ONE* **15**, e0233848 (2020).

15. E. Ferrante, A. E. Turgut, E. Duéñez-Guzmán, M. Dorigo, T. Wenseleers, Evolution of self-organized task specialization in robot swarms. *PLoS Comput. Biol.* **11**, e1004273 (2015).

16. M. Waibel, D. Floreano, L. Keller, A quantitative test of Hamilton's rule for the evolution of altruism. *PLoS Biol.* **9**, e1000615 (2011).

17. M. Waibel, L. Keller, D. Floreano, Genetic team composition and level of selection in the evolution of cooperation. *IEEE Trans. Evol. Comput.* **13**, 648–660 (2009).

18. A. F. Winfield, Evolutionary robotics as a modelling tool in evolutionary biology. *Front. Robot. AI* **11**, 1278983 (2024).

19. N. Kanwisher, M. Khosla, K. Dobs, Using artificial neural networks to ask 'why'questions of minds and brains. *Trends Neurosci.* **46**, 240–254 (2023).

20. B. Cheung, E. Weiss, B. A. Olshausen, Emergence of foveal image sampling from learning to attend in visual scenes. arXiv:1611.09430 (2016).

21. R. Pramod, H. Katti, S. Arun, Human peripheral blur is optimal for object recognition. *Vision Res.* **200**, 108083 (2022).

22. D. Silver, T. Hubert, J. Schrittwieser, I. Antonoglou, M. Lai, A. Guez, M. Lanctot, L. Sifre, D. Kumaran, T. Graepel, T. Lillicrap, K. Simonyan, D. Hassabis, Mastering chess and shogi by self-play with a general reinforcement learning algorithm. arXiv:1712.01815 (2017).

23. A. Fawzi, M. Balog, A. Huang, T. Hubert, B. Romera-Paredes, M. Barekatain, A. Novikov, F. J. R. Ruiz, J. Schrittwieser, G. Swirszcz, D. Silver, D. Hassabis, P. Kohli, Discovering faster matrix multiplication algorithms with reinforcement learning. *Nature* **610**, 47–53 (2022).

24. D. Silver, T. Hubert, J. Schrittwieser, I. Antonoglou, M. Lai, A. Guez, M. Lanctot, L. Sifre, D. Kumaran, T. Graepel, T. Lillicrap, K. Simonyan, D. Hassabis, A general reinforcement

learning algorithm that masters chess, shogi, and go through self-play. *Science* **362**, 1140–1144 (2018).

25. D. J. Mankowitz, A. Michi, A. Zhernov, M. Gelmi, M. Selvi, C. Paduraru, E. Leurent, S. Iqbal, J.-B. Lespiau, A. Ahern, T. Köppe, K. Millikin, S. Gaffney, S. Elster, J. Broshear, C. Gamble, K. Milan, R. Tung, M. Hwang, T. Cemgil, M. Barekatain, Y. Li, A. Mandhane, T. Hubert, J. Schrittwieser, D. Hassabis, P. Kohli, M. Riedmiller, O. Vinyals, D. Silver, Faster sorting algorithms discovered using deep reinforcement learning. *Nature* **618**, 257–263 (2023).

26. D.-E. Nilsson, The evolution of eyes and visually guided behaviour. *Philos. Trans. R. Soc. Lond. B. Biol. Sci.* **364**, 2833–2847 (2009).

27. E. Warrant, D.-E. Nilsson, *Invertebrate Vision* (Cambridge Univ. Press, 2006).

28. D.-E. Nilsson, The evolution of visual roles—Ancient vision versus object vision. *Front. Neuroanat.* **16**, 789375 (2022).

29. D.-E. Nilsson, S. Pelger, A pessimistic estimate of the time required for an eye to evolve. *Proc. Roy. Soc. London Ser. B. Biol. Sci.* **256**, 53–58 (1994).

30. J. Kaplan, S. McCandlish, T. Henighan, T. B. Brown, B. Chess, R. Child, S. Gray, A. Radford, J. Wu, D. Amodei, Scaling laws for neural language models. arXiv:2001.08361 (2020).

31. E. M. Caves, N. C. Brandley, S. Johnsen, Visual acuity and the evolution of signals. *Trends Ecol. Evol.* **33**, 358–372 (2018).

32. M. V. Srinivasan, S. Zhang, M. Lehrer, T. Collett, Honeybee navigation en route to the goal: Visual flight control and odometry. *J. Exp. Biol.* **199**, 237–244 (1996).

33. E. Todorov, T. Erez, Y. Tassa, "MuJoCo: A physics engine for model-based control" in *IROS* (IEEE, 2012), pp. 5026–5033.

34. W. J. Gehring, The evolution of vision. *WIREs Dev. Biol.* **3**, 1–40 (2014).

35. W. J. Gehring, K. Ikeo, Pax 6: Mastering eye morphogenesis and eye evolution. *Trends Genet.* **15**, 371–377 (1999).

36. G. K. Aguirre, A model of the entrance pupil of the human eye. *Sci. Rep.* **9**, 9360 (2019).

37. M. Doebeli, I. Ispolatov, Chaos and unpredictability in evolution. *Evolution* **68**, 1365–1373 (2014).

38. D. C. O'Shea, "Monochromatic quartet: a search for the global optimum" in *1990 Intl Lens Design Conf*, G. N. Lawrence, Ed. (International Society for Optics; Photonics; SPIE, 1991), vol. 1354, pp. 548–554; https://doi.org/10.1117/12.47896.

39. C. Gagné, J. Beaulieu, M. Parizeau, S. Thibault, Human-competitive lens system design with evolution strategies. *Appl. Soft Comput.* **8**, 1439–1452 (2008).

40. A. Gupta, S. Savarese, S. Ganguli, L. Fei-Fei, Embodied intelligence via learning and evolution. *Nat. Commun.* **12**, 5721 (2021).

41. K. Sims, "Artificial evolution for computer graphics," in *Proceedings of the 18th Annual Conference on Computer Graphics and Interactive Techniques* (Association for Computing Machinery, 1991), pp. 319–328; https://doi.org/10.1145/122718.122752.

42. D. Floreano, T. Kato, D. Marocco, E. Sauser, Coevolution of active vision and feature selection. *Biol. Cybern.* **90**, 218–228 (2004).

43. D.-E. Nilsson, The diversity of eyes and vision. *Annu. Rev. Vis. Sci.* **7**, 19–41 (2021).

44. M. Towers, A. Kwiatkowski, J. Terry, J. U. Balis, G. De Cola, T. Deleu, M. Goulão, A. Kallinteris, M. Krimmel, A. KG, R. Perez-Vicente, A. Pierre, S. Schulhoff, J. J. Tai, H. Tan, O. G. Younis, Gymnasium: A standard interface for reinforcement learning environments. arXiv:2407.17032 (2024).

45. T. Baden, From water to land: Evolution of photoreceptor circuits for vision in air. *PLoS Biol.* **22**, e3002422 (2024).

46. J. M. Baldwin, A new factor in evolution. *Am. Nat.* **30**, 441–451 (1896).

47. K. O. Stanley, R. Miikkulainen, Evolving neural networks through augmenting topologies. *Evol. Comput.* **10**, 99–127 (2002).

48. T. Salimans, J. Ho, X. Chen, S. Sidor, I. Sutskever, Evolution strategies as a scalable alternative to reinforcement learning.arXiv:1703.03864 (2017).

49. E. Conti, V. Madhavan, F. P. Such, J. Lehman, K. O. Stanley, J. Clune, Improving exploration in evolution strategies for deep reinforcement learning via a population of novelty-seeking agents. arXiv:171206560 (2018).

50. N. Hansen, A. Ostermeier, "Adapting arbitrary normal mutation" in *Proceedings of the 1996 IEEE International Conference on Evolutionary Computation* (1996), pp. 312–317.

51. J. Rapin, O. Teytaud, Nevergrad—A gradient-free optimization platform, GitHub Repository (2018); https://github.com/facebookresearch/nevergrad.

52. J. Schulman, F. Wolski, P. Dhariwal, A. Radford, O. Klimov, Proximal policy optimization algorithms. arXiv:1707.06347 (2017).

53. R. F. Burton, The scaling of eye size in adult birds: Relationship to brain, head and body sizes. *Vision Res.* **48**, 2345–2351 (2008).

54. M. de L. Brooke, S. Hanley, S. Laughlin, The scaling of eye size with body mass in birds. *Proc. R. Soc. Lond. Ser. B. Biol. Sci.* **266**, 405–412 (1999).

55. E. K. Buschbeck, B. Ehmer, R. R. Hoy, Chunk versus point sampling: Visual imaging in a small insect. *Science* **286**, 1178–1180 (1999).

56. R. Wehner, Desert ant navigation: How miniature brains solve complex tasks. *J. Comp. Physiol. A* **189**, 579–588 (2003).

57. W. R. Jeffery, Regressive evolution in astyanax cavefish. *Annu. Rev. Genet.* **43**, 25–47 (2009).

58. C. Hogg, M. Neveu, K. Stokkan, L. Folkow, P. Cottrill, R. Douglas, D. Hunt, G. Jeffery, Arctic reindeer extend their visual range into the ultraviolet. *J. Exp. Biol.* **214**, 2014–2019 (2011).

59. I. R. Schwab, The evolution of eyes: Major steps. The Keeler lecture 2017: Centenary of Keeler Ltd. *Eye* **32**, 302–313 (2018).

60. D.-E. Nilsson, Eye evolution and its functional basis. *Vis. Neurosci.* **30**, 5–20 (2013).

61. J. N. P. Martel, L. K. Müller, S. J. Carey, P. Dudek, G. Wetzstein, Neural sensors: Learning pixel exposures for HDR imaging and video compressive sensing with programmable sensors. *IEEE Trans. Pattern Anal. Mach. Intell.* **42**, 1642–1653 (2020).

62. Y. Wu, V. Boominathan, H. Chen, A. Sankaranarayanan, A. Veeraraghavan, "Phasecam3d—Learning phase masks for passive single view depth estimation" in *2019 IEEE International Conference on Computational Photography (ICCP)* (IEEE, 2019), pp. 1–12.

63. V. Sitzmann, S. Diamond, Y. Peng, X. Dun, S. Boyd, W. Heidrich, F. Heide, G. Wetzstein, End-to-end optimization of optics and image processing for achromatic extended depth of field and super-resolution imaging. *ACM Trans. Graph.* **37**, 1–13 (2018).

64. C. H. Huang, M. J. Zhong, W. B. Liao, A. Kotrschal, Investigating the role of body size, ecology, and behavior in anuran eye size evolution. *Evol. Ecol.* **33**, 585–598 (2019).

65. L. Z. Garamszegi, A. P. Møller, J. Erritzøe, Coevolving avian eye size and brain size in relation to prey capture and nocturnality. *Proc. R. Soc. Lond. Ser. B. Biol. Sci.* **269**, 961–967 (2002).

66. A. Corral-López, M. Garate-Olaizola, S. D. Buechel, N. Kolm, A. Kotrschal, On the role of body size, brain size, and eye size in visual acuity. *Behav. Ecol. Sociobiol.* **71**, 1–10 (2017).

67. R. Burton, A new look at the scaling of size in mammalian eyes. *J. Zool.* **269**, 225–232 (2006).

68. S. Herculano-Houzel, The human brain in numbers: A linearly scaled-up primate brain. *Front. Hum. Neurosci.* **3**, 31 (2009).

69. U. Dicke, G. Roth, Neuronal factors determining high intelligence. *Philos. Trans. R. Soc. B Biol. Sci.* **371**, 20150180 (2016).

70. S. Herculano-Houzel, The remarkable, yet not extraordinary, human brain as a scaled-up primate brain and its associated cost. *Proc. Natl. Acad. Sci. U.S.A.* **109**, 10661–10668 (2012).

71. C. Enroth-Cugell, J. G. Robson, The contrast sensitivity of retinal ganglion cells of the cat. *J. Physiol.* **187**, 517–552 (1966).

72. S. W. Kuffler, Discharge patterns and functional organization of mammalian retina. *J. Neurophysiol.* **16**, 37–68 (1953).

73. T. Gollisch, M. Meister, Eye smarter than scientists believed: Neural computations in circuits of the retina. *Neuron* **65**, 150–164 (2010).

74. J. Hoffmann, S. Borgeaud, A. Mensch, E. Buchatskaya, T. Cai, E. Rutherford, D. de Las Casas, L. A. Hendricks, J. Welbl, A. Clark, T. Hennigan, E. Noland, K. Millican, G. van den Driessche, B. Damoc, A. Guy, S. Osindero, K. Simonyan, E. Elsen, J. W. Rae, O. Vinyals, L. Sifre, Training compute-optimal large language models. arXiv:2203.15556 (2022).

75. X. Zhai, A. Kolesnikov, N. Houlsby, L. Beyer, Scaling vision transformers. *Proc. IEEE/CVF Conf. Comput. Vis. Pattern Recognit.* , 12104–12113 (2022).

76. M. A. Gordon, K. Duh, J. Kaplan, Data and parameter scaling laws for neural machine translation. *Proc. 2021 Conf. Empir. Methods Nat. Lang. Process.* , 5915–5922 (2021).

77. I. M. Alabdulmohsin, B. Neyshabur, X. Zhai, Revisiting neural scaling laws in language and vision. *Adv. Neural Inf. Process. Syst.* **35**, 22300–22312 (2022).

78. K. Shinomiya, A. Nern, I. A. Meinertzhagen, S. M. Plaza, M. B. Reiser, Neuronal circuits integrating visual motion information in *Drosophila melanogaster*. *Curr. Biol.* **32**, 3529–3544.e2 (2022).

79. J. Lehman, J. Clune, D. Misevic, *C. Adami*, L. Altenberg, J. Beaulieu, P. J. Bentley, S. Bernard, G. Beslon, D. M. Bryson, P. Chrabaszcz, N. Cheney, A. Cully, S. Doncieux, F. C. Dyer, K. O. Ellefsen, R. Feldt, S. Fischer, S. Forrest, A. Frénoy, C. Gagné, L. L. Goff, L. M. Grabowski, B. Hodjat, F. Hutter, L. Keller, C. Knibbe, P. Krcah, R. E. Lenski, H. Lipson, R. MacCurdy, C. Maestre, R. Miikkulainen, S. Mitri, D. E. Moriarty, J.-B. Mouret, A. Nguyen, C. Ofria, M. Parizeau, D. Parsons, R. T. Pennock, W. F. Punch, T. S. Ray, M. Schoenauer, E. Shulte, K. Sims, K. O. Stanley, F. Taddei, D. Tarapore, S. Thibault, W. Weimer, R. Watson, J. Yosinski, The surprising creativity of digital evolution: A collection of anecdotes from the evolutionary computation and artificial life research communities. arXiv: 1803.03453 (2019).

80. T. Klinghoffer, K. Tiwary, N. Behari, B. Agrawalla, R. Raskar, "DISeR: Designing imaging systems with reinforcement learning" in *Proceedings of the IEEE/CVF International Conference on Computer Vision* (2023), pp. 23632–23642.

81. K. Tiwary, T. Klinghoffer, A. Young, S. Somasundaram, N. Behari, A. Dave, B. Cheung, D.-E. Nilsson, T. Poggio, R. Raskar, "A Roadmap for Generative Design of Visual Intelligence. An MIT Exploration of Generative AI" (2024); https://www.researchgate.net/publication/384121135_A_Roadmap_for_Generative_Design_of_Visual_Intelligence.

82. A. Raffin, A. Hill, A. Gleave, A. Kanervisto, M. Ernestus, N. Dormann, Stable-baselines3: Reliable reinforcement learning implementations. *J. Mach. Learn. Res.* **22**, 1–8 (2021).

83. P. Henderson, R. Islam, P. Bachman, J. Pineau, D. Precup, D. Meger, "Deep reinforcement learning that matters" in *Proceedings of the AAAI Conference on Artificial Intelligence* (2018), vol. 32.

84. N. Hansen, A. Ostermeier, Completely derandomized self-adaptation in evolution strategies. *Evol. Comput.* **9**, 159–195 (2001).

85. G. Holló, M. Novák, The manoeuvrability hypothesis to explain the maintenance of bilateral symmetry in animal evolution. *Biol. Direct* **7**, 22 (2012).

86. Z. Tasneem, G. Milione, Y.-H. Tsai, X. Yu, A. Veeraraghavan, M. Chandraker, F. Pittaluga, "Learning Phase Mask for Privacy-Preserving Passive Depth Estimation" in *European Conference on Computer Vision* (Springer, 2022), pp. 504–521.

87. J. Chang, G. Wetzstein, "Deep optics for monocular depth estimation and 3D object detection" in *Proceedings of the IEEE/CVF International Conference on Computer Vision* (2019), pp. 10193–10202.

88. J. Chang, G. Wetzstein, Deep optics for monocular depth estimation and 3D object detection. arXiv: 1904.08601 (2019).

89. J. W. Goodman, *Introduction to Fourier Optics* (Roberts and Company Publishers, 2005).

90. M. Woo, J. Neider, T. Davis, D. Shreiner, *OpenGL Programming Guide: The Official Guide to Learning OpenGL, Version 1.2* (Addison-Wesley Longman Publishing Co., Inc., 1999).

91. E. Adelson, J. Bergen, "The plenoptic function and the elements of early vision" (1997); https://www.researchgate.net/publication/2684713_The_Plenoptic_Function_and_the_Elements_of_Early_Vision.

92. N. J. Marshall, A unique colour and polarization vision system in mantis shrimps. *Nature* **333**, 557–560 (1988).

93. M. Land, Structure of the retinae of the principal eyes of jumping spiders (salticidae: Dendryphantinae) in relation to visual optics. *J. Exp. Biol.* **51**, 443–470 (1958).

94. "Allometry: The study of biological scaling" (2018); https://api.semanticscholar.org/CorpusID:199531454.

95. C. Venditti, J. Baker, R. A. Barton, Co-evolutionary dynamics of mammalian brain and body size. *Nat. Ecol. Evol.* **8**, 1534–1542 (2024).

96. J. Hestness, S. Narang, N. Ardalani, G. Diamos, H. Jun, H. Kianinejad, Md. M. A. Patwary, Y. Yang, Y. Zhou, Deep learning scaling is predictable, empirically. arXiv:1712.00409 (2017).
